# Exponential growth, high prevalence of SARS-CoV-2, and vaccine effectiveness associated with the Delta variant

**DOI:** 10.1126/science.abl9551

**Published:** 2021-12-17

**Authors:** Paul Elliott, David Haw, Haowei Wang, Oliver Eales, Caroline E. Walters, Kylie E. C. Ainslie, Christina Atchison, Claudio Fronterre, Peter J. Diggle, Andrew J. Page, Alexander J. Trotter, Sophie J. Prosolek, The COVID-19 Genomics UK (COG-UK) Consortium, Deborah Ashby, Christl A. Donnelly, Wendy Barclay, Graham Taylor, Graham Cooke, Helen Ward, Ara Darzi, Steven Riley

**Affiliations:** 1 School of Public Health, Imperial College London, London, UK.; 2 Imperial College Healthcare NHS Trust, London, UK.; 3 National Institute for Health Research Imperial Biomedical Research Centre, London, UK.; 4 MRC Centre for Environment and Health, School of Public Health, Imperial College London, London, UK.; 5 Health Data Research UK London at Imperial College London, London, UK.; 6 UK Dementia Research Institute Centre at Imperial, London, UK.; 7 MRC Centre for Global Infectious Disease Analysis and Jameel Institute, Imperial College London, London, UK.; 8 Centre for Infectious Disease Control, National Institute for Public Health and the Environment, Bilthoven, Netherlands.; 9 CHICAS, Lancaster Medical School, Lancaster University, and Health Data Research UK, Lancaster, UK.; 10 Quadram Institute, Norwich, UK.; 11 www.cogconsortium.uk.; 12 Department of Statistics, University of Oxford, Oxford, UK.; 13 Department of Infectious Disease, Imperial College London, London, UK.; 14 Institute of Global Health Innovation at Imperial College London, London, UK.; 15 Health Security Initiative, Flagship Pioneering UK Ltd., Bristol, UK.

## Abstract

Severe acute respiratory syndrome coronavirus 2 (SARS-CoV-2) infections were rising during early summer 2021 in many countries as a result of the Delta variant. We assessed reverse transcription polymerase chain reaction swab positivity in the Real-time Assessment of Community Transmission–1 (REACT-1) study in England. During June and July 2021, we observed sustained exponential growth with an average doubling time of 25 days, driven by complete replacement of the Alpha variant by Delta and by high prevalence at younger, less-vaccinated ages. Prevalence among unvaccinated people [1.21% (95% credible interval 1.03%, 1.41%)] was three times that among double-vaccinated people [0.40% (95% credible interval 0.34%, 0.48%)]. However, after adjusting for age and other variables, vaccine effectiveness for double-vaccinated people was estimated at between ~50% and ~60% during this period in England. Increased social mixing in the presence of Delta had the potential to generate sustained growth in infections, even at high levels of vaccination.

## Vaccination and disease

The United Kingdom has high rates of vaccination for severe acute respiratory syndrome coronavirus 2 (SARS-CoV-2), exceeding 80% of adults. As immunity wanes and social distancing is relaxed, how are rates of illness and severe disease affected by more infectious variants? Elliott *et al*. used reverse transcription PCR data from the REACT-1 study, which showed exponential transmission as the Alpha variant (B.1.1.7) was replaced by the Delta variant (B.1.617.2). After adjusting for age and other variables, vaccine effectiveness for the new variant averaged 55% in June and July of 2020. Despite the slower growth of the pandemic in the summer, it looks as if increased indoor mixing in the autumn will sustain transmission of the Delta variant despite high levels of adult vaccination. —CA

## Abstract (structured)

**BACKGROUND:**

The prevalence of severe acute respiratory syndrome coronavirus 2 (SARS-CoV-2) infection continues to drive rates of illness and hospitalizations despite high levels of vaccination, with the proportion of cases caused by the Delta lineage increasing in many populations. As vaccination programs roll out globally and social distancing is relaxed, future SARS-CoV-2 trends are uncertain.

**METHODS:**

The Real-time Assessment of Community Transmission–1 (REACT-1) study has been tracking the spread of the COVID-19 pandemic in England since May 2020. The study involves obtaining a self-administered throat and nose swab for reverse transcription polymerase chain reaction (RT-PCR) from ~100,000 or more people during 2 to 3 weeks each month, based on random samples of the population in England at ages 5 years and above. As well as information on swab positivity, we collect demographic and other data on potential risk factors and (since January 2021) vaccination history. Prevalence estimates are weighted to be representative of the population of England as a whole. Here, we analyzed prevalence trends and their drivers using RT-PCR swab positivity data from REACT-1 round 12 (between 20 May and 7 June 2021) and round 13 (between 24 June and 12 July 2021). Response rates, defined as the percentage of invitees from whom we received a valid swab result, were 20.4% across all rounds and 13.4% and 11.7% for rounds 12 and 13, respectively.

**RESULTS:**

We observed sustained exponential growth as the third wave in England took hold, with reproduction number *R* estimated at 1.44 (95% credible interval 1.20, 1.73) in round 12 and 1.19 (1.06, 1.32) in round 13, corresponding to an average doubling time of 11 days (7, 23 days) in round 12 and 25 days (15, >50 days) in round 13. This resulted in an increase in average weighted prevalence from 0.15% (0.12%, 0.18%) in round 12 (based on 135 positives out of 108,911 valid swabs) to 0.63% (0.57%, 0.69%) in round 13 (527 positives out of 98,233). The rapid growth across and within rounds appears to have been driven by complete replacement of the Alpha variant by Delta, and by the high prevalence in younger, less-vaccinated age groups: Among those aged 13 to 17 years, we observed an increase in weighted prevalence by a factor of 9 between round 12 [0.16% (0.08%, 0.31%)] and round 13 [1.56% (1.25%, 1.95%)]. In round 13, weighted prevalence among those who reported being unvaccinated [1.21% (1.03%, 1.41%)] was greater than for those who reported having had two doses of vaccine [0.40% (0.34%, 0.48%)] by a factor of 3; however, 44% of infections occurred in doubly vaccinated individuals, reflecting imperfect vaccine effectiveness (VE) against infection after two doses despite high overall levels of vaccination.

Among participants aged 18 to 64 years, on the basis of self-reported vaccination status, we estimated VE against infection (adjusted for age, sex, region, ethnicity, and index of multiple deprivation) of 49% (95% confidence interval 22%, 67%) in round 13, rising to 58% (33%, 73%) when only strong positives [cycle threshold (Ct) values below 27] were considered. For the same age group, we estimated adjusted VE of 59% (23%, 78%) against symptomatic infection—that is, among those reporting one or more common COVID-19 symptoms in the month prior to testing (fever, loss or change of sense of smell or taste, new persistent cough). Ethnicity, household size, and local levels of deprivation, in addition to age, jointly contributed to the risk of higher prevalence of swab positivity.

**CONCLUSION:**

From the end of May to the beginning of July 2021 in England, where there was a highly successful vaccination campaign with high vaccine uptake, infections were increasing exponentially—driven by the Delta variant—with high infection prevalence among younger, unvaccinated individuals. Despite slower growth (or level or declining prevalence) during summer 2021 in the Northern Hemisphere, increased mixing in the presence of the Delta variant likely explains renewed growth that occurred in autumn 2021, even in populations with high levels of vaccination.

## Teaser

The SARS-CoV-2 Delta variant increased exponentially in the United Kingdom driven by infection in the young, not yet vaccinated, population.

## Graphical abstract

**Figure:**
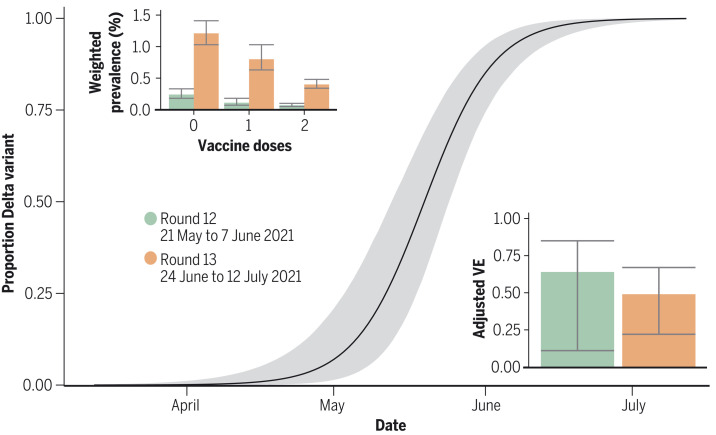
During 2021, SARS-CoV-2 variant replacement caused a rise in infections and raised concerns about vaccine effectiveness (VE) against infection. Main and top left: Complete replacement of Alpha by the Delta variant from REACT-1 round 12 to round 13 and weighted prevalence of SARS-CoV-2 infection among a random sample of the population of England ages 5 years and above by self-reported vaccine status. Bottom right: VE adjusted for age, sex, index of multiple deprivation, region, and ethnicity.

Despite the successful development, licensing, and distribution of effective vaccines against COVID-19 (*1*, *2*), the number of newly reported cases and deaths continued to rise globally into the Northern Hemisphere summer of 2021 (*3*). Prior trends of decreasing prevalence were being reversed in some populations where the Delta variant had become dominant, leading to estimates of a substantially higher transmissibility for Delta relative to Alpha (*4*). In addition, globally, as of July 2021, only 13% of the population were double-vaccinated and only 1% of people in low-income countries had received even one dose (*5*). Despite slower growth (or level or declining prevalence) during the Northern Hemisphere summer, many countries experienced a further large wave of infections in the autumn, driven by the Delta variant.

The vaccine rollout in England started with the oldest and most vulnerable groups, beginning in December 2020. Since then, there has been a strong correlation among age, vaccine type, and date of vaccination, with individuals receiving the same vaccine for first and second dose. Initially, health care workers and older adults received BNT162b2 before doses were switched to ChAdOx1 for many people between the ages of 40 and 80 and some younger people. The program then switched back to BNT162b2 for those below the age of 40 (also using small numbers of mRNA-1273 vaccine). Subsequently, from September 2021, the vaccination program was expanded to include children from the age of 12 years.

The incidence of reverse transcription polymerase chain reaction (RT-PCR)–confirmed cases of COVID-19 increased substantially in England after the Delta variant became established during April and May 2021 (*6*). Over the same period, the UK government proceeded with its gradual relaxation of social distancing (roadmap out of lockdown) (*7*) and the ending of almost all legal restrictions in England on 19 July 2021 (*8*). Although a much lower proportion of COVID-19 cases resulted in hospitalizations in England versus a comparable period of growth during autumn 2020, exponential growth in hospitalizations was still observed from mid-June 2021 (*6*).

With first data collection starting in May 2020, we established the Real-time Assessment of Community Transmission–1 (REACT-1) study to track the spread of the COVID-19 pandemic in England and improve situational awareness (*9*, *10*). The study involves obtaining a self-administered throat and nose swab for RT-PCR from ~100,000 or more people during 2 to 3 weeks each month, based on random samples of the population in England at ages 5 years and above (see materials and methods). As well as information on swab positivity, we collect demographic and contextual data including (since January 2021) on vaccination history. By July 2021, ~1.9 million people had taken part (table S1). Here, we describe the key patterns of severe acute respiratory syndrome coronavirus 2 (SARS-CoV-2) infections for round 12 (20 May to 7 June 2021) and round 13 (24 June to 12 July 2021) during the third wave of the epidemic in England. Valid RT-PCR results were obtained from 108,911 participants in round 12 and 98,233 participants in round 13 (table S1).

## Prevalence and growth

**Fig. 1.**
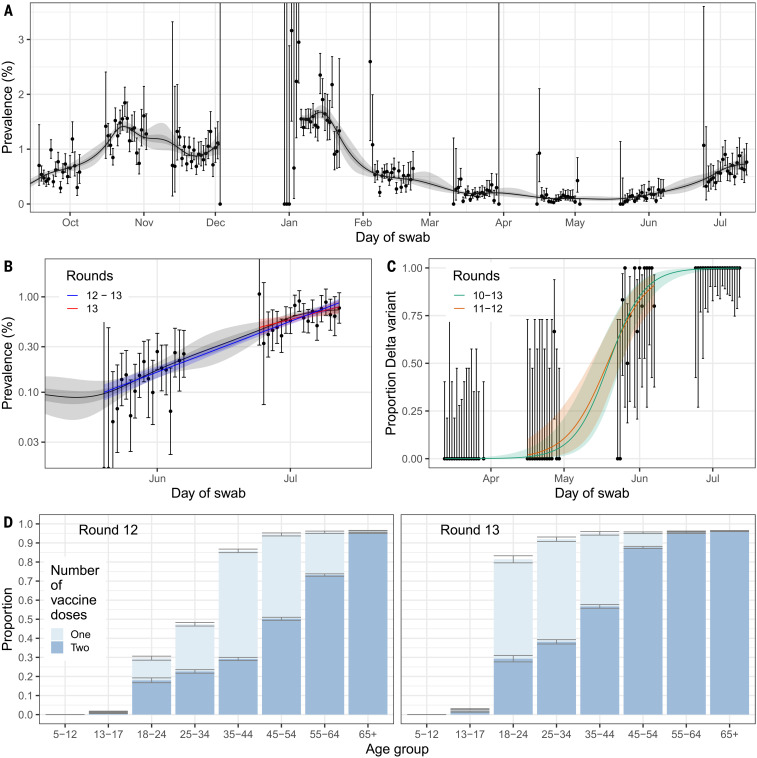
Temporal trends in prevalence, proportion of positive cases determined to be the Delta variant, and vaccine coverage. (**A**) Prevalence of national swab positivity for England estimated using a P-spline for all 13 rounds with central 50% (dark gray) and 95% (light gray) posterior credible intervals. From round 5 of the study onward, weighted observations (black dots) and 95% binomial confidence intervals (vertical lines) are also shown. Note that the period between rounds 7 and 8 (December) of the model is not included, as there were no data available to capture the late December peak of the epidemic. (**B**) Comparison of the exponential model fit to round 12 and 13 (blue) and the exponential model fit to round 13 only (red). Also shown is the P-spline model fit from (A). Shown here only for rounds 12 and 13 of the study with a log_10_ *y* axis. (**C**) Proportion of Delta against Alpha over time. Points show raw data; error bars denote the 95% confidence interval. Shaded regions show best-fit Bayesian logistic regression models, fit to rounds 10 to 13 (green) and rounds 11 and 12 (orange), with 95% credible interval. (**D**) Proportion of individuals with known vaccine status who reported being vaccinated with one (light blue) or two (dark blue) doses. Error bars denote 95% binomial confidence intervals.

Prevalence of infection with SARS-CoV-2 increased substantially in England between rounds 12 and 13 (Fig. 1) as the third wave took hold, linked to the rapid replacement of Alpha by the Delta variant. In round 13, between 24 June and 12 July 2021, we found 527 positives from 98,233 swabs, giving a weighted prevalence of 0.63% [95% credible interval (CrI) 0.57%, 0.69%], and, on average, a factor of >4 rise relative to the weighted prevalence in round 12 of 0.15% (CrI 0.12%, 0.18%) (table S1). The prevalence in round 13 was similar to that observed in early October 2020 and late January 2021 during, respectively, the rise and fall of the second wave (Fig. 1).

The Delta variant completely replaced Alpha during the period of our study, consistent with genomic data from outbreak investigation and routine surveillance (*11*). Of the 254 lineages determined for round 13, 100% were the Delta variant, compared with round 12 during which 36 of 46 (78.3%) were Delta and the remaining 10 were the Alpha variant. The growth of Delta against Alpha from round 10 (11 to 30 March 2021) to round 13 corresponded to a daily growth rate advantage of 0.14 (CrI 0.10, 0.20) for Delta, which, in turn, implied an additive *R* advantage of 0.86 (CrI 0.63, 1.23) (Fig. 1). This is consistent with estimates based on trends in the proportion of positive PCR assays where the S gene was not detected [presumed to be Alpha (*12*)] and on differences in household attack rate for households where Delta was identified rather than Alpha (*13*). Within the Delta variant, we did not detect the K417N mutation associated with the AY.1 and AY.2 lineages. Under the assumption that REACT-1 participants provide an unbiased sample of infections, we can exclude, with 95% confidence, a population prevalence of non-Delta lineages greater than 0.004%, corresponding to 2350 infections in England on average during round 13.

Nationally, we observed an exponential trend in prevalence with sustained growth for rounds 12 and 13 (between 20 May and 12 July 2021) (Fig. 1 and table S2) despite England having one of the highest adult vaccination rates internationally (*5*). Averaging over the period of each of rounds 12 and 13 separately, we estimated the reproduction number *R* at 1.44 (CrI 1.20, 1.73) (round 12) and 1.19 (CrI 1.06, 1.32) (round 13), corresponding to doubling times of 11 days (CrI 7, 23 days) and 25 days (15, >50 days) respectively. Across rounds 12 and 13, *R* was 1.28 (CrI 1.24, 1.31) with a doubling time of 17 days (CrI 15, 19 days). Patterns of growth for the period of the study were robust when considering alternative definitions of positivity, such as only nonsymptomatic individuals or positive samples with lower cycle threshold (Ct) values, corresponding to higher viral load (table S2).

## Age

Alongside the rapid rise of the Delta variant, recent growth in England appears to have been driven by younger age groups (table S3 and fig. S1). For example, in 13- to 17-year-olds, weighted prevalence in round 13 [1.56% (CrI 1.25%, 1.95%)] was higher than in round 12 [0.16% (CrI 0.08%, 0.31%)] by a factor of 9. Similar patterns were observed in England for the same period in a longitudinal household study (*14*). In contrast, at ages 65 to 74 years, weighted prevalence increased from round 12 [0.07% (CrI 0.04%, 0.12%)] to round 13 [0.25% (CrI 0.19%, 0.34%)] by a factor of 3 to 4. More generally, participants aged between 5 and 24 years were overrepresented among infected people in our study, contributing 50% of infections (weighted age-standardized) while only representing 25% of the population of England aged 5 years or above (*15*). Therefore, whether because of mixing patterns, infectiousness or susceptibility, this group was driving transmission and, during a period of exponential growth, any vaccination targeted at the younger ages would have a disproportionate impact in slowing the epidemic (*16*).

## Prevalence among vaccinated and unvaccinated

Participants who reported having received two doses of vaccine were at substantially reduced risk of testing positive relative to those who reported not being vaccinated. For round 13, the prevalence of swab positivity among unvaccinated participants [1.21% (CrI 1.03%, 1.41%)] was greater for all ages than among those who had received two doses of vaccine [0.40% (CrI 0.34%, 0.48%)] by a factor of 3 (table S3). The prevalence in unvaccinated relative to double-vaccinated individuals was similar for round 12, with a prevalence of 0.24% (CrI 0.18%, 0.33%) in those unvaccinated versus 0.07% (CrI 0.05%, 0.10%) in those reporting two doses (table S3).

However, these estimates conflate the effect of vaccination with other correlated variables such as age, which is strongly associated with likelihood of having been vaccinated and also acts as a proxy for differences in behavior across the age groups. Specifically, in England, few children and young people under the age of 18 years have been vaccinated twice, whereas few over the age of 65 years remain unvaccinated (Table 1 and Fig. 1). We therefore restricted the analyses to those aged 18 to 64 years (*n* = 64,415 in round 12, *n* = 57,457 in round 13), which permitted direct contrast of infection rates between double-vaccinated and unvaccinated groups (Table 1).

**Table 1.** Self-reported and linked vaccination status and swab positivity in rounds 12 and 13 of REACT-1 shown for all participants (5 years and above) and for the subset aged 18 to 64 years.

**Dataset**	**Age group**	**Vaccine status**	**Round 12**			**Round 13**		
			**Negative**	**Positive**	**Odds ratio**	**Negative**	**Positive**	**Odds ratio**
Self- reported	All	Unvaccinated	22,709	51	Reference	14,957	178	Reference
		Vaccinated (1 dose)	18,654	20	0.48 (0.28, 0.80)	9,598	77	0.67 (0.52, 0.88)
		Vaccinated (2 or more doses)	48,383	30	0.28 (0.18, 0.43)	55,765	197	0.30 (0.24, 0.36)
		Vaccinated (unknown doses)	2,889	1	0.15 (0.02, 1.12)	3,314	11	0.28 (0.15, 0.51)
		Vaccine status not known	16,141	33	0.91 (0.59, 1.41)	14,072	64	0.38 (0.29, 0.51)
	18–64	Unvaccinated	9,012	16	Reference	2,574	28	Reference
		Vaccinated (1 dose)	18,307	19	0.58 (0.30, 1.14)	9,467	76	0.74 (0.48, 1.14)
		Vaccinated (2 or more doses)	25,248	17	0.38 (0.19, 0.75)	34,503	145	0.39 (0.26, 0.58)
		Vaccinated (unknown doses)	1,173	0	0.00 (0.00, NA)	1,517	9	0.55 (0.26, 1.16)
		Vaccine status not known	10,597	26	1.38 (0.74, 2.58)	9,089	49	0.50 (0.31, 0.79)
Linked	All	Unvaccinated	19,115	52	Reference	11,357	153	Reference
		Vaccinated (1 dose)	26,285	33	0.46 (0.30, 0.71)	11,885	93	0.58 (0.45, 0.75)
		Vaccinated (2 or more doses)	50,721	34	0.25 (0.16, 0.38)	61,202	206	0.25 (0.20, 0.31)
	18–64	Unvaccinated	8,099	21	Reference	1,553	25	Reference
		Vaccinated (1 dose)	25,657	32	0.48 (0.28, 0.83)	11,652	92	0.49 (0.31, 0.77)
		Vaccinated (2 or more doses)	23,511	18	0.30 (0.16, 0.55)	36,448	153	0.26 (0.17, 0.40)
	All	Unvaccinated	19,115	52	Reference	11,357	153	Reference
		Vaccinated (<14 days 2nd dose)	31,826	35	0.40 (0.26, 0.62)	13,425	102	0.56 (0.44, 0.73)
		Vaccinated (≥14 days 2nd dose)	45,180	32	0.26 (0.17, 0.40)	59,662	197	0.25 (0.20, 0.30)
	18–64	Unvaccinated	8,099	21	Reference	1,553	25	Reference
		Vaccinated (<14 days 2nd dose)	30,593	34	0.43 (0.25, 0.74)	13,170	101	0.48 (0.31, 0.74)
		Vaccinated (≥14 days 2nd dose)	18,575	16	0.33 (0.17, 0.64)	34,930	144	0.26 (0.17, 0.39)

At these ages, we compared swab-negatives with (i) all swab-positives and (ii) the subset of swab-positives who were symptomatic [i.e., reporting one or more common COVID-19 symptoms in the month prior to testing (fever, loss or change of sense of smell or taste, new persistent cough)]. After adjusting for age, sex, region, ethnicity, and index of multiple deprivation (IMD) (*17*), for all swab-positives, we estimated vaccine effectiveness (VE) of 64% [95% confidence interval (CI) 11%, 85%] in round 12 and 49% (CI 22%, 67%) in round 13 among people who had received two doses of vaccine of any type. For those with symptoms, we estimated VE of 83% (CI 19%, 97%) in round 12 and 59% (CI 23%, 78%) in round 13 (Table 2).

**Table 2.** Unadjusted and adjusted estimates of vaccine effectiveness against infection for self-reported vaccine status and linked vaccine status for rounds 12 and 13 of REACT-1 for participants aged 18 to 64 years.

**Vaccination data source (*n*)**	**Adjustment**	**Vaccine effectiveness (2 doses)**	
		**Round 12**	**Round 13**
Self-report, all positives, 18 to 64 years	Age, sex	61% (2%, 84%)	47% (18%, 65%)
	Age, sex, IMD, region, ethnicity	64% (11%, 85%)	49% (22%, 67%)
Self-report, symptomatic only, 18 to 64 years	Age, sex	81% (5%, 96%)	56% (19%, 77%)
	Age, sex, IMD, region, ethnicity	83% (19%, 97%)	59% (23%, 78%)
Linked, all positives, 18 to 64 years	Age, sex	75% (33%, 90%)	61% (36%, 76%)
	Age, sex, IMD, region, ethnicity	75% (35%, 90%)	62% (38%, 77%)

Independent data on vaccination status was provided for 57,338 (89%) participants aged 18 to 64 in round 12 consenting to data linkage, and 49,923 (87%) in round 13 (materials and methods). Using these linked data, we estimated adjusted VE at 75% (CI 35%, 90%) in round 12 and 62% (CI 38%, 77%) in round 13. The apparently higher VE for the linked participants reflected differences in odds of infection among the linked and unlinked groups (table S4), suggesting possible bias introduced by consent to linkage, but also some misclassification of vaccine status in the self-reported data (table S5). Because reported dates of vaccination were more reliable in the linked data, we used those data to examine the effect of including a lag period of 14 days after the second vaccination and observed similar odds ratios for zero lag and 14-day lag following the second dose (Table 1). In addition, we observed a similar unweighted prevalence of swab positivity among double-vaccinated individuals who did and did not report prior infection more than 28 days before their swab (table S5), which suggests in our study that prior infection did not materially affect the estimate of VE. Moreover, the strong correlation among age, vaccine type, and time since vaccination in England, together with limited numbers, prevented us from being able to reliably assess the impact of vaccine type or time since infection independently of age.

Although vaccination was associated with lower prevalence of swab positivity, there remained potential for large numbers of people who had received two doses of vaccine to become infected. During the period of round 12, we extrapolated from our data that 29% of infections in England occurred in double-vaccinated people, rising to 44% during the period of round 13. These increases in prevalence in vaccinated individuals in round 13 could be driven by increased social mixing or by a higher proportion of infections being the Delta variant, or attributable to waning of protection from infection. Also, although lower than for unvaccinated individuals, nearly one in 25 double-vaccinated individuals [3.84% (CI 2.81%, 5.21%)] tested swab-positive if they reported contact with a known COVID-19 case (table S6).

## Cycle threshold values

**Fig. 2.**
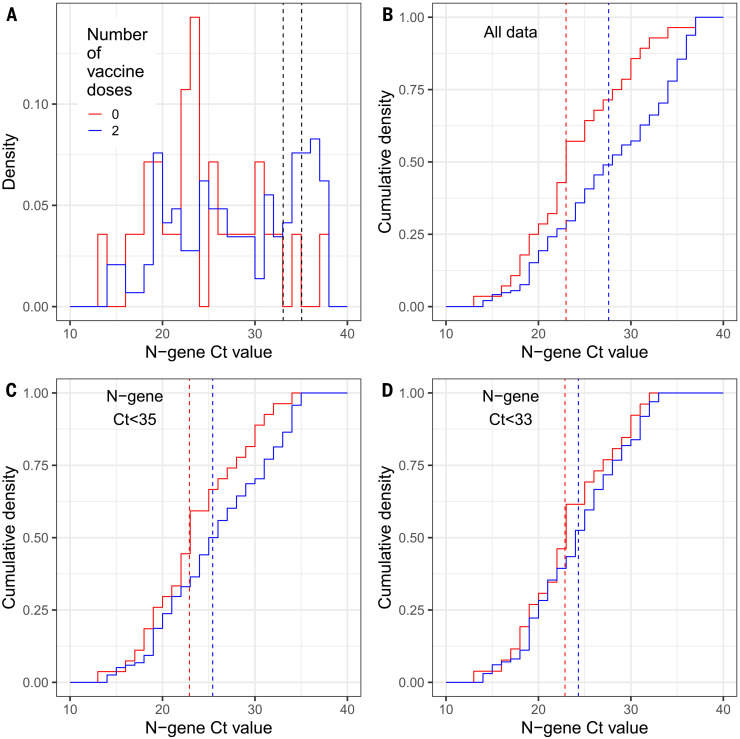
Distribution of N-gene Ct values, by vaccine status, for positive samples obtained from individuals aged 18 to 64 years inclusive. (**A**) Distribution of all N-gene Ct values for those who are unvaccinated (red) and those who reported receiving two doses of a vaccine (blue). Also shown are two black dashed lines at N-gene Ct = 33 and 35; these show the threshold values for a sample to be classed as positive, used in sensitivity analyses. (**B**) Cumulative density of N-gene Ct values using all available data for unvaccinated individuals (red) and individuals who have had two doses of a vaccine (blue). (**C**) Cumulative density of N-gene Ct values using all data in which N-gene Ct is less than 35 for unvaccinated individuals (red) and individuals who have had two doses of a vaccine (blue). (**D**) Cumulative density of N-gene Ct values using all data in which N-gene Ct is less than 33 for unvaccinated individuals (red) and individuals who have had two doses of a vaccine (blue). In (B) to (D), red and blue vertical dashed lines show the median value for each distribution.

We analyzed Ct values associated with positive results among vaccinated and unvaccinated individuals as a measure of viral load. For all positives in round 13, at ages 18 to 64 years, median Ct value for vaccinated participants [27.6 (CI for median, 25.5, 29.7)] was higher than for unvaccinated ones [23.1 (CI 20.3, 25.8) (positive defined as N gene Ct below 37 or both N gene– and E gene–detected; see materials and methods) (Fig. 2 and table S7). The higher Ct values among vaccinated people may suggest lower infectiousness (*18*), consistent with transmission studies conducted when the Alpha variant was dominant, in which vaccinated individuals were at substantially lower risk of passing on infection (*19*). As a secondary analysis, we reduced the Ct threshold for positivity to capture strong positives, which resulted in a smaller difference in median Ct values between vaccinated and unvaccinated individuals (Fig. 2, C and D). At the same time, our estimate of VE for those who reported having received two doses of vaccine increased to 54% (CI 29%, 71%) for a Ct threshold of 35, plateauing between 57% (CI 32%, 72%) and 58% (CI 33%, 73%) for a Ct threshold of 33 and 27, respectively.

## Time series of infections, hospital admissions, and deaths

**Fig. 3.**
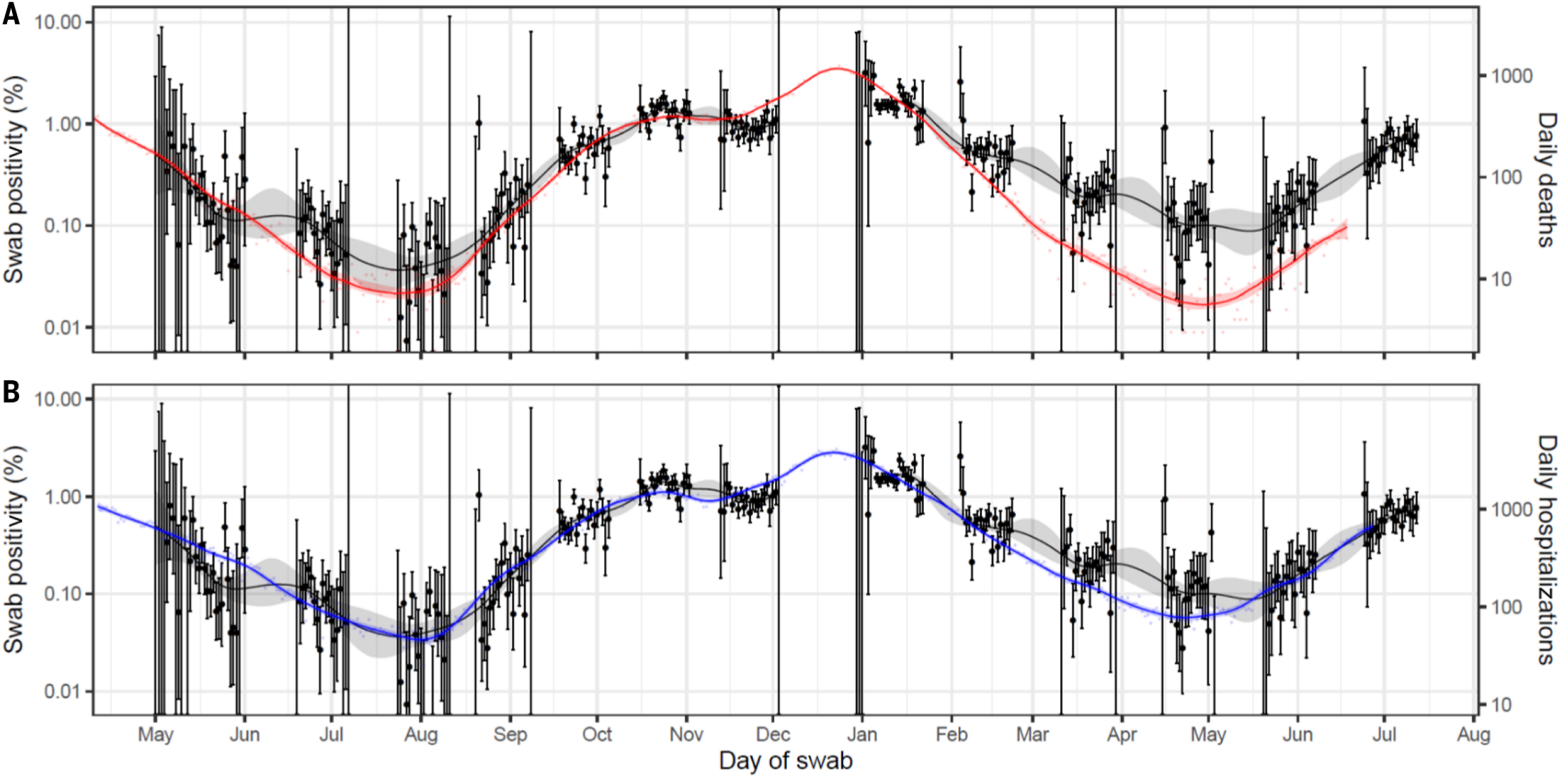
Comparison of daily deaths and hospitalizations to swab positivity as measured by REACT-1. Daily swab positivity for all 13 rounds of the REACT-1 study (black points with 95% confidence intervals, left *y* axis) with P-spline estimates for swab positivity (solid black line; shaded area is 95% credible interval). (**A**) Daily deaths in England (red points, right *y* axis) and P-spline model estimates for expected daily deaths in England (solid red line, right *y* axis; shaded area is 95% credible interval). Daily deaths have been shifted by 26 (26, 26) days backward in time along the *x* axis. The two *y* axes have been scaled using the best-fit population adjusted scaling parameter 0.059 (0.058, 0.061). (**B**) Daily hospitalizations in England (blue points, right *y* axis) and P-spline model estimates for expected daily hospitalizations in England (solid blue line, right *y* axis; shaded area is 95% credible interval). Daily hospitalizations have been shifted by 20 (19, 20) days backward in time along the *x* axis. The two *y* axes have been scaled using the best-fit population adjusted scaling parameter 0.241 (0.236, 0.246).

We next investigated how swab positivity measured in REACT-1 related to daily hospital admissions and deaths in publicly available data (*6*), finding a best-fitting lag between swab positivity and hospitalizations of 20 days and between swab positivity and deaths of 26 days (Fig. 3). At these lags, from early February 2021, there was a clear divergence between swab positivity and deaths, coinciding with the rollout of England’s mass vaccination campaign, with a smaller divergence between swab positivity and hospitalizations. However, as the Delta variant became dominant in mid-April 2021, the associations between infections and hospitalizations and deaths began to reconverge, both for people below and above 65 years (fig. S2).

## Geographical variation

At the regional level, estimates of *R* were consistent with the overall trend within round 13. Prevalence in round 13 was highest in London at 0.94% (CrI 0.76%, 1.16%), up from 0.13% (CrI 0.08%, 0.20%) in round 12 (table S3). There was a suggestion of a possible slowing of the rise in London in the most recent data, although with wide confidence intervals (table S8).

At the subregional level, there was a suggestion of prevalence of infection decreasing in some areas and increasing in others (fig. S3). For example, in the North West of England, high prevalence in a large urban area covering Greater Manchester and Lancashire during the first half of round 13 was less evident in the second half, whereas prevalence increased between the first and second halves in nearby south Yorkshire, part of the Yorkshire and The Humber region. These data are indicative of rapidly changing local spread of the virus within the context of the national exponential rise in infections.

## Ethnicity, household size, and neighborhood deprivation

Ethnicity, household size, and area levels of deprivation jointly contributed to the risk of higher prevalence of swab positivity, in addition to age. Unadjusted prevalence (table S3) showed highest prevalence in people of Black ethnicity at 1.21% (CrI 0.75%, 1.93%) compared with 0.59% (CrI 0.53%, 0,65%) in people of white ethnicity; highest prevalence in those in the largest households of six or more people at 1.35% (CrI 0.90%, 2.01%) compared with 0.44% (CrI 0.32%, 0.61%) and 0.44% (CrI 0.36%, 0.53%) in single- and two-person households, respectively; and highest prevalence in participants living in the most deprived neighborhoods at 0.82% (CrI 0.65%, 1.04%) compared with the least deprived at 0.48% (CrI 0.39%, 0.59%). Prior rounds of REACT-1 have shown different ethnicities at increased prevalence at different times, consistently higher prevalence of infection in larger households, and usually increased prevalence in more deprived neighborhoods (*20*–*25*). In models including each of the above variables, similar patterns were observed in the odds of testing positive, although odds were reduced when all three of the above variables were considered jointly, together with age, sex, region, and key worker status (table S9). Age remained an important predictor of swab positivity in these mutually adjusted models. Also, in these analyses, women had lower odds of infection than men at 0.80 (CI 0.67, 0.96) in round 13, although not in round 12 at 1.34 (CI 0.93, 1.92) (table S9); this difference may be related to increased social mixing associated with England’s progression in the Euro 2020 football competition during June and July 2021, as was seen previously in Scottish data, reflecting their earlier exit from the competition (*26*).

## Discussion

We report a rapidly rising prevalence of infection in England during 20 May to 12 July 2021 associated with the replacement of Alpha by the Delta variant in a highly vaccinated population. Our central estimate of VE against all SARS-CoV-2 infections for two doses of vaccine (self-report) was 49% in the most recent data, increasing to 58% when we defined effectiveness only for strong positives, and 62% in the linked data. These estimates are lower than some others (*19*, *27*, *28*) but consistent with more recent data from Israel (*29*).

Estimates of VE are not absolute but will vary depending on a variety of factors. Our estimates were higher when we restricted our analyses to people reporting symptoms of COVID-19 in the previous month and to those who consented to linkage of health records, although still lower than those from routine testing of symptomatic people presenting for RT-PCR in England (*27*). Unlike routine testing, our data are based on a random sample of the population and include asymptomatic people, as well as symptomatic individuals who may not present for testing; our results may therefore give a less biased representation of infection risk. Also, our estimated effectiveness was lower than that from a longitudinal household survey that included asymptomatic individuals but was conducted before the emergence of Delta, where vaccine status was based on a mix of self-reported and linked data (*19*). More generally, estimates of VE may depend on vaccine type, interval between doses, possible waning over time, and the extent of past natural infection among the comparator (unvaccinated) group.

We show that the third wave of infections in England was being driven primarily by the Delta variant in younger, unvaccinated people. This focus of infection offers considerable scope for interventions to reduce transmission among younger people, with knock-on benefits across the entire population. Also, given the rapid rise of the Delta variant that occurred in Europe, the US, South Asia and elsewhere, and its estimated increased transmissibility, patterns in England were informative of what was subsequently observed elsewhere. In our data, the highest prevalence of infection during June to July 2021 was among 13- to 24-year-olds. In the UK, the Joint Committee on Vaccinations and Immunizations recommended in August 2021 that vaccination should be offered to all 16- and 17-year-olds and then in September 2021 further extended the UK program to include children aged 12 to 15 years, as has been done in the US and some other countries. This expansion of the vaccination program to those at highest risk of infection had the potential to reduce transmission in the autumn and winter 2021 as levels of social mixing, including indoors, increased (*30*). Also, development of vaccines against Delta and other variants may be warranted in the light of evidence of antigenic change measured by neutralization (*31*) and the relationship between neutralization titer and protection from mild disease (*32*).

Estimates of VE against serious outcomes of greater than 90% have been reported for those who have received two doses of either BNT162b2 (*33*) or ChAdOx1-S (*34*) vaccines. This is in keeping with our observation of a weakening of the association between infections and hospitalizations and deaths from mid-February to early April 2021 when the Alpha variant was dominant. However, in our more recent data (since mid-April 2021), infections and hospitalizations began to reconverge, potentially reflecting the increased prevalence and severity of Delta compared with Alpha (*35*), a changing age mix of hospitalized cases to younger ages, and possible waning of protection (*29*, *36*).

Our study has limitations. One estimate of effectiveness was based on self-reported vaccine status, because we could only obtain linked vaccination data for the subset of participants who gave consent, with individuals who did and did not consent to linkage appearing to have different patterns of swab positivity across the vaccinated and unvaccinated groups. Because age, date of vaccination, and vaccine type are so strongly correlated in England, and with limitations in numbers, we were wary of introducing a time variable into the analyses to investigate the waning of VE explicitly. However, the design of the study—based on estimation of infection prevalence from independent samples within (as well as across) separate rounds, conducted monthly—itself provides strong control for any time effects.

Over the course of the study since round 1 in May 2020, toward the end of the first lockdown in England, we observed a gradual reduction in response rates, from 30.5% in round 1 to 11.7% in round 13. These rates are conservative estimates because they are based on numbers of swabs with a valid RT-PCR result compared to the total number of letters of invitation sent out, some of which may have been returned, sent to the wrong address, or left unopened by the recipient. Nonetheless, the drop in response rates means that our sample may be becoming less representative, particularly in some groups such as young people (18 to 24 years) and those living in the most deprived areas where response rates by round 13 had fallen to 4.2% and 5.1%, respectively. Note, however, that these response rates have been achieved without the use of financial or other incentives.

Our method of sampling was designed initially to achieve sufficient numbers in each lower-tier local authority (LTLA) in England so that we could analyze subregional trends and also, by weighting the sample, provide estimates of prevalence that were representative of the population of England. Whereas previously we had aimed to achieve approximately equal numbers of people in our sample by LTLA, in rounds 12 and 13 we switched to sampling in proportion to population in order to capture greater resolution in inner-city areas, which were relatively underrepresented in our previous sampling regimen. In either case, as we reweight the sample according to the national population profile, weighted prevalence should be comparable across rounds, albeit with lower precision in later rounds because of the lower response rates.

Our data show that rapid exponential growth of SARS-CoV-2 prevalence occurred during the third wave in England at a time when the Delta variant became dominant. The rapid rollout of the vaccination program in England has so far limited the number of infections and serious cases relative to the unvaccinated population. Level or declining prevalence was observed during summer 2021 in the Northern Hemisphere, reflecting school vacations, greater time spent outdoors, and reduced social interactions. But without additional interventions, increased mixing (including indoors) in the presence of the Delta variant likely explains renewed growth that occurred in autumn 2021, even in populations with high levels of vaccination. Continued surveillance to monitor the spread of the epidemic is therefore required.

## Materials and methods

The REACT-1 study methods have been described elsewhere (*9*). Briefly, at each round, we sent an invitation by post to named individuals from the list of patients registered with a National Health Service (NHS) general practitioner in England, obtained from NHS Digital, covering almost the entire population. We included all 317 LTLAs in England, and by combining the Isles of Scilly with Cornwall and the City of London with Westminster, we report results across 315 LTLAs overall.

For round 1 to round 11, we aimed to obtain approximately equal numbers of participants in each LTLA to be powered to provide local estimates of prevalence. From round 12 onward, we adjusted the sampling procedure to select the sample randomly in proportion to population at the LTLA level, thus obtaining more samples in LTLAs with higher population density in inner urban areas. However, we ensured that data were comparable across rounds as we reweighted the data at each round to be representative of England as a whole (see below).

For those registering to participate, we obtained age, sex, address, and residential postcode from the NHS register and collected additional information on demographics, health, and lifestyle via online or telephone questionnaire. This included information on ethnicity, smoking, household size, key worker status, contact with a known or suspected COVID-19 case, and whether, at time of survey, participants had experienced one or more of 29 symptoms in the past week or past month (participants not reporting symptoms may have developed symptoms later, but these were not captured). Participants were also asked for consent to longer-term follow-up through linkage to their NHS records including data from the national immunization program. The questionnaires are available on the study website (*37*).

Response rates have varied by age and over time and place, and are available for each round [“For Researchers: REACT-1 Study Materials” (*37*)]. Overall response rate was defined as the percentage of invitees from whom we received a valid swab result; this was 20.4% across all rounds, and 13.4% and 11.7% for rounds 12 and 13, respectively. In round 13, response rate varied by age from 4.2% at ages 18 to 24 years to 24% at ages 65 to 74 years and by IMD decile from 5.1% in the most deprived areas to 20.8% in the least deprived.

Participants were requested to provide a self-administered throat and nose swab (obtained by parent or guardian for children aged 5 to 12 years) following written and video instructions. Swabs were placed into a dry tube (no solution or preservative), refrigerated at home, picked up by courier, and then sent chilled to a single commercial laboratory for testing for SARS-CoV-2 by RT-PCR.

### Ct threshold and laboratory calibration experiments

We tested two gene targets (the E and N genes) with Ct values used as a proxy for intensity of viral load. The RT-PCR test was considered positive if both gene targets were detected or if the N gene was detected with a Ct value less than 37. The Ct threshold used to determine positivity was set following three separate calibration experiments. First, 10 RNA extraction plates were sent from the commercial laboratory for blinded reanalysis in two laboratories accredited by the UK Accreditation Service (UKAS). We found concordant results for 919 negative samples and all 40 controls. We detected viral RNA in 11 of the 19 samples with a Ct value reported positive by the commercial laboratory (N gene Ct value ranging from 16.5 to 40.7); in 10 of these 11 samples, the N gene Ct value was <37. Second, in a serial dilution experiment of synthetic SARS-CoV-2 RNA, the commercial laboratory detected 2.5 copies at Ct 38; also while following serial dilution of known positive samples with low viral load, the commercial laboratory identified an N gene signal at Ct > 37 in most instances. Third, a Public Health England (PHE) reference laboratory reanalyzed a further 40 unblinded positive samples (on 19- × 96-well plates) with N gene Ct values > 35 (range 35.7 to 46.8) and without a signal for an E gene, detecting SARS-CoV-2 RNA in 15/40 (38%) samples (2/4 with N gene Ct value < 37). The results of all three calibration experiments were then consolidated to set the positivity criteria noted above, which have been used throughout each round of REACT-1.

### Prevalence estimates and weighting

We obtained unweighted (crude) prevalence estimates for different sociodemographic and occupational groups by dividing counts of swab positivity (based on RT-PCR) by the number of swabs returned in that group. We then applied rim weighting (*38*) to provide prevalence weighted to be representative of the population of England as a whole, by age, sex, deciles of the IMD, LTLA counts, and ethnic group. We obtained the age by sex and LTLA counts from the Office for National Statistics mid-year population estimates (*39*) and counts by ethnic group from the Labour Force Survey (*40*), and calculated the IMD decile points from linkage of postcode to area-level IMD using the original sampling frame obtained from NHS Digital. Because of the different sources of population estimates, the rim weighting was based on proportions rather than population totals. We grouped age into nine categories: 5 to 12; 13 to 17; 18 to 24; 25 to 34; 35 to 44; 45 to 54; 55 to 64; 65 to 74; 75 years or above, giving 18 age-sex categories. Self-reported ethnicity was grouped into nine categories: white; mixed/multiple ethnic groups; Indian; Pakistani; Bangladeshi; Chinese; any other Asian background; Black African/Caribbean/other; and any other ethnic group or missing.

For the rim weighting, initially (first stage) the sample was weighted to LTLA counts and age by sex groups only, adjusting the age and sex groups to ensure that the final weighted estimates were as close as possible to the population profile. Then, using the first-stage weights as starting weights, the rim weighting was adjusted for all four measures, with the adjustment factor between the first- and second-stage weights trimmed at the 1st and 99th percentiles to dampen the extreme weights and improve efficiency. The final weights were calculated as the first-stage weights multiplied by the trimmed adjustment factor for the second stage, with confidence intervals for weighted prevalence estimates calculated using the “survey” package in R (*41*).

### Statistical analyses

Statistical analyses were carried out in R (*42*). To investigate the potential confounding effects of covariates on prevalence estimates, we performed logistic regression on swab positivity as the outcome, and sex, age, region, employment type, ethnicity, household size, and neighborhood deprivation as explanatory variables. We adjusted for age and sex, and mutually adjusted for the other covariates to obtain odds ratio estimates and 95% confidence intervals. We decided not to adjust for multiple testing to facilitate direct comparisons with other publications where only comparison-wise error rate (CER) has been controlled for (*43*).

We estimated adjusted VE as 1 – (odds ratio) where the odds ratio was obtained from comparing vaccinated and unvaccinated individuals in a logistic regression model with swab positivity as outcome and with adjustment for age and sex, and age, sex, IMD quintile, and ethnicity.

To estimate the underlying geographical variation in prevalence at the local (subregional) level, we used a neighborhood spatial smoothing method based on nearest neighbor up to 30 km. We calculated *N_n_*, the median number of study participants within 30 km of each study participant for each round or subround. We then calculated the local prevalence for 15 members of each LTLA as an estimate of the smoothed neighborhood prevalence in that area.

To analyze trends in swab positivity over time, we used an exponential model of growth or decay with the assumption that the weighted number of positive samples (from the weighted total number of samples) each day arose from a binomial distribution. The model is of the form (*t*) = *I*_0_.*e*, where *I*(*t*) is the swab positivity at time *t*, *I*_0_ is the swab positivity on the first day of data collection per round, and *r* is the growth rate. The binomial likelihood for *P* (out of *N*) positive tests on a given day is then *P* ~ (*N*, *I*_0_.*e^rt^*) based on day of swabbing or, if unavailable, day of sample collection. We used a bivariate No-U-Turn sampler to estimate posterior credible intervals assuming uniform prior distributions on *I*_0_ and *r* (*44*). We estimated the reproduction number *R* assuming a generation time that follows a gamma distribution with a shape parameter, *n*, of 2.29 and a rate parameter, β, of 0.36 (corresponding to a mean generation time of 6.29 days) (*45*). *R* was estimated from the equation *R* = (1 + *r*/β)^*n* (*46*) using data from two sequential rounds and separately per round. We carried out a range of sensitivity analyses including estimation of *R* for different thresholds of Ct values that determine swab positivity and for nonsymptomatic individuals (not reporting symptoms on the day of swab or month prior).

We fit a Bayesian penalized spline (P-spline) model (*47*) to the daily data using a No-U-Turn Sampler in logit space, segmenting the data into approximately 5-day sections by regularly spaced knots, with further knots beyond the study period to minimize edge effects. We defined fourth-order basis splines (b-splines) over the knots with the final model consisting of a linear combination of these b-splines. We guarded against overfitting by including a second-order random-walk prior distribution on the coefficients of the b-splines, taking the form *b_i_* = 2*b_i_*_–1_ – *b_i_*_–2_ + *u_i_*, where* b_i_* is the *i*th b-spline coefficient and *u_i_* is normally distributed with *u_i_* ~ *N*(0, ρ^2^). This prior penalizes against changes in the growth rate unless supported by the data; the strength of the penalization is determined by the parameter ρ for which we assume an inverse gamma prior distribution, ρ ~ *IG*(0.001, 0.001). We assume that the first two b-spline coefficients have uniform distribution (i.e., *b*_1_ and *b*_2_ ~ constant).

We compared daily prevalence data from rounds 1 to 13 of REACT-1 with publicly available national daily hospital admissions and COVID-19 mortality data (deaths within 28 days of a positive test). To do this, we fit P-spline models as before to the daily hospital admissions and to the daily death data in order to obtain estimates for the expected number of outcomes on a given day. We then fit a simple two-parameter model consisting of a lag time between the posterior of the P-spline estimate for each of hospitalizations or deaths, the daily weighted prevalence calculated from REACT-1 data, and a scaling parameter, corresponding to the percentage of people who were swab-positive in the population on a particular day in comparison with future hospitalizations or deaths. Because of the time delay between the REACT-1 prevalence signal and daily hospitalizations and deaths, the model was only fit to rounds 1 to 12. We then compared round 13 data to the estimated trend in hospitalizations and deaths to visualize any alterations in the link between these parameters and infection prevalence as measured in REACT-1. We estimated these relationships for all ages and separately for those aged under 65 years, and those 65 years and above.

To visualize the trends of the REACT-1 data over time, we also fitted P-splines to all subsets of the REACT-1 data examined. For the REACT-1 data split by age (below 65 years and 65 years and above), we fit a mixed P-spline model in which a P-spline was fit separately to each age group but the smoothing parameter, ρ, was fit to both datasets simultaneously. Further changes in the first derivative were assumed to happen at the same time for both datasets, with the condition *u_i_*_,<65_ – *u_i_*_,65+_ ~ *N*(0, η^2^) and η given an uninformative prior distribution, η ~ *IG*(0.001, 0.001).

### Viral genome sequencing

RT-PCR positive swab samples where there was sufficient sample volume and with N gene Ct values of <32 were sent frozen from the laboratory to the Quadram Institute (Norwich, UK) for viral genome sequencing. Amplification of viral RNA used the ARTIC protocol (*48*) and sequencing libraries were prepared using CoronaHiT (*49*). Analysis of sequencing data used the ARTIC bioinformatic pipeline (*50*) with lineages assigned using PangoLEARN (*51*).

We fit a Bayesian logistic regression model to the proportion of lineages that were identified as the Delta variant from round 10 to round 13 to obtain a daily growth rate advantage between Delta and other circulating lineages, Δ*r*. Assuming an exponential generation time of mean 6.29 days (*45*), the reproduction number, *R*, is given by $R=1+r\times \underline{g}$ (*46*). The estimate of growth rate advantage can thus be converted into an additive *R* advantage through the equation $\Delta R=\Delta r\times \underline{g}$, assuming the mean generation time is the same for all lineages. We chose not to estimate a multiplicative *R* advantage (*52*), because it relies on the assumption of a zero-variance discrete generation time interval, which is less consistent with estimates of an overdispersed serial interval (*45*).

As a sensitivity the model was also fit to data from only round 11 to round 12 to check that edge effects were not introducing bias. The upper bound of prevalence for non-Delta lineages (none of which were detected in round 13) was estimated by calculating the 95% Wilson upper bound on the proportion of non-Delta lineage detected, then multiplying by the weighted prevalence estimate for round 13. This was then multiplied by the population of England to get an estimate for the upper bound on the average number of people infected with a non-Delta lineage at any one time during round 13.

### Data availability

Access to REACT-1 individual-level data is restricted to protect participants’ anonymity. Summary statistics, descriptive tables, and code from the current REACT-1 study are available at https://github.com/mrc-ide/reactidd. REACT-1 study materials are available for each round at www.imperial.ac.uk/medicine/research-and-impact/groups/react-study/react-1-study-materials/.

### Public involvement

A Public Advisory Panel provides input into the design, conduct, and dissemination of the REACT research program.

### Ethics

We obtained research ethics approval from the South Central–Berkshire B Research Ethics Committee (IRAS ID: 283787).

## Supplementary Material

20211102-1

## Supplementary Materials

### Other Supplementary Material for this manuscript includes the following:

Data S1

COG-UK Consortium member list

MDAR Reproducibility Checklist
