# Supplementary material for: Exponential growth, high prevalence of SARS-CoV-2, and vaccine effectiveness associated with the Delta variant
Source: Science. 2021 Dec 17;374(6574):eabl9551. doi: 10.1126/science.abl9551 (PMC10763627; doi:10.1126/science.abl9551)
Supplement: Supplementary file 2 — Materials and Methods Tables S1 to S9 Figs. S1 to S3 [file science.abl9551_sm.pdf]

Science AAAS

Supplementary Materials for

Exponential growth, high prevalence SARS-CoV-2, and vaccine effectiveness associated with the Delta variant

Paul Elliott *et al.*

Corresponding authors: Paul Elliott, p.elliott@imperial.ac.uk; Steven Riley, s.riley@imperial.ac.uk

Science **374**, eabl9551 (2021)
DOI: 10.1126/science.abl9551

The PDF file includes:

Materials and Methods
Tables S1 to S9
Figs. S1 to S3

Other Supplementary Material for this manuscript includes the following:

Data S1
COG-UK Consortium member list
MDAR Reproducibility Checklist

Table S1. Unweighted (95% confidence interval) and weighted (95% credible interval) prevalence of swab positivity across 13 rounds of REACT-1.

Round	Tested swabs	Positive swabs	Unweighted prevalence (95% CI) *	Weighted prevalence (95% CI)	First sample	Last sample
1	120,620	159	0.13% (0.11%, 0.15%)	0.16% (0.13%, 0.19%)	1/5/2020	1/6/2020
2	159,199	123	0.077% (0.065%, 0.092%)	0.088% (0.068%, 0.11%)	19/6/2020	7/7/2020
3	162,821	54	0.033% (0.025%, 0.043%)	0.040% (0.027%, 0.053%)	24/7/2020	11/8/2020
4	154,325	137	0.089% (0.075%, 0.11%)	0.13% (0.096%, 0.15%)	20/8/2020	8/9/2020
5	174,949	824	0.47% (0.44%, 0.50%)	0.60% (0.55%, 0.71%)	18/9/2020	5/10/2020
6	160,175	1,732	1.08% (1.03%, 1.13%)	1.30% (1.21%, 1.39%)	16/10/2020	2/11/2020
7	168,181	1,299	0.77% (0.73%, 0.82%)	0.94% (0.87%, 1.01%)	13/11/2020	3/12/2020
8	167,642	2,282	1.36% (1.31%, 1.42%)	1.57% (1.49%, 1.66%)	06/01/2021*	22/01/2021
9	165,456	689	0.42% (0.39%, 0.45%)	0.49% (0.44%, 0.55%)	4/2/2021	23/2/2021
10	140,844	227	0.16% (0.14%, 0.18%)	0.20% (0.17%, 0.23%)	11/03/2021	30/3/2021
11	127,408	115	0.09% (0.07%, 0.11%)	0.10% (0.08%, 0.13%)	15/04/2021	3/5/2021
12	108,911	135	0.12% (0.10%, 0.15%)	0.15% (0.12%, 0.18%)	20/05/2021	07/06/2021
13	98,233	527	0.54% (0.49%, 0.58%)	0.63% (0.57%, 0.69%)	24/06/2021	12/07/2021

* Sampling strategy changed for round 12 and subsequent rounds. Therefore unweighted prevalence is not directly comparable with previous rounds

Table S2. Estimates of national growth rates, doubling times and reproduction numbers for round 13, round 12 to round 13, and round 12.

Round	Outcome	Growth rate	R	Probability R>1	Doubling (+) / Halving (-) time
13	All positives	0.028 (0.009 , 0.046)	1.19 (1.06 , 1.32)	>0.99	25.0 (* , 15.1)
	Non-symptomatics	0.042 (0.011 , 0.072)	1.28 (1.07 , 1.52)	>0.99	16.7 (* , 9.6)
	Positive for both E and N genes	0.019 (-0.002 , 0.041)	1.13 (0.99 , 1.28)	0.96	35.7 (* , 16.9)
	Positive for both E and N genes or positive only for N gene with CT 35 or less	0.030 (0.011 , 0.050)	1.20 (1.07 , 1.35)	>0.99	23.2 (* , 13.9)
12 and 13	All positives	0.041 (0.036 , 0.046)	1.28 (1.24 , 1.31)	>0.99	17.0 (19.2 , 15.2)
	Non-symptomatics	0.042 (0.034 , 0.050)	1.29 (1.23 , 1.35)	>0.99	16.6 (20.5 , 13.8)
	Positive for both E and N genes	0.042 (0.036 , 0.048)	1.29 (1.25 , 1.33)	>0.99	16.5 (19.1 , 14.5)
	Positive for both E and N genes or positive only for N gene with CT 35 or less	0.041 (0.036 , 0.046)	1.28 (1.24 , 1.32)	>0.99	17.0 (19.4 , 15.1)
12	All positives	0.063 (0.030 , 0.097)	1.44 (1.20 , 1.73)	>0.99	11.1 (23.4 , 7.1)
	Non-symptomatics	0.030 (-0.027 , 0.087)	1.20 (0.84 , 1.64)	0.85	23.3 (-26.0 , 8.0)
	Positive for both E and N genes	0.104 (0.064 , 0.146)	1.79 (1.45 , 2.18)	>0.99	6.7 (10.8 , 4.8)
	Positive for both E and N genes or positive only for N gene with CT 35 or less	0.073 (0.037 , 0.110)	1.53 (1.25 , 1.84)	>0.99	9.5 (18.7 , 6.3)

* Doubling/Halving time had an estimated magnitude greater than 50 days and so represented approximately constant prevalence

Table S3a. Unweighted (95% confidence interval) and weighted (95% credible interval) prevalence of swab-positivity for sex, age, and region for round 12 and round 13.

Variable	Category	Round 12				Round 13			
		Positive	Total	Unweighted Prevalence	Weighted Prevalence*	Positive	Total	Unweighted Prevalence	Weighted Prevalence*
Gender	Male	55	48,190	0.11% (0.09% , 0.15%)	0.14% (0.10% , 0.18%)	252	43,272	0.58% (0.51% , 0.66%)	0.71% (0.62% , 0.82%)
	Female	80	60,718	0.13% (0.10% , 0.16%)	0.16% (0.12% , 0.20%)	275	54,957	0.50% (0.44% , 0.56%)	0.55% (0.48% , 0.63%)
	Unknown	0	3	0.00% (0.00% , 70.76%)	NA (NA , NA)	0	4	0.00% (0.00% , 60.24%)	0 (NA , NA)
Age	05-12	26	7,598	0.34% (0.22% , 0.50%)	0.35% (0.23% , 0.54%)	70	6,994	1.00% (0.78% , 1.26%)	1.02% (0.80% , 1.31%)
	13-17	10	5,906	0.17% (0.08% , 0.31%)	0.16% (0.08% , 0.31%)	86	5,423	1.59% (1.27% , 1.95%)	1.56% (1.25% , 1.95%)
	18-24	12	4,044	0.30% (0.15% , 0.52%)	0.36% (0.20% , 0.64%)	47	3,117	1.51% (1.11% , 2.00%)	1.56% (1.15% , 2.13%)
	25-34	11	9,059	0.12% (0.06% , 0.22%)	0.11% (0.06% , 0.21%)	54	7,888	0.68% (0.51% , 0.89%)	0.72% (0.54% , 0.97%)
	35-44	16	12,592	0.13% (0.07% , 0.21%)	0.12% (0.07% , 0.21%)	73	11,876	0.61% (0.48% , 0.77%)	0.61% (0.48% , 0.78%)
	45-54	23	17,564	0.13% (0.08% , 0.20%)	0.14% (0.09% , 0.22%)	74	15,776	0.47% (0.37% , 0.59%)	0.46% (0.36% , 0.58%)
	55-64	16	21,156	0.08% (0.04% , 0.12%)	0.08% (0.05% , 0.14%)	59	18,800	0.31% (0.24% , 0.40%)	0.31% (0.24% , 0.40%)
	65-74	15	20,647	0.07% (0.04% , 0.12%)	0.07% (0.04% , 0.12%)	48	18,836	0.25% (0.19% , 0.34%)	0.25% (0.19% , 0.34%)
	75+	6	10,345	0.06% (0.02% , 0.13%)	0.07% (0.03% , 0.18%)	16	9,523	0.17% (0.10% , 0.27%)	0.17% (0.10% , 0.29%)
Region	South East	21	18,855	0.11% (0.07% , 0.17%)	0.14% (0.09% , 0.23%)	61	17,427	0.35% (0.27% , 0.45%)	0.39% (0.29% , 0.51%)
	North East	6	5,018	0.12% (0.04% , 0.26%)	0.14% (0.05% , 0.34%)	27	4,500	0.60% (0.40% , 0.87%)	0.74% (0.48% , 1.14%)
	North West	22	13,229	0.17% (0.10% , 0.25%)	0.26% (0.16% , 0.41%)	81	11,814	0.69% (0.54% , 0.85%)	0.77% (0.59% , 0.99%)
	Yorkshire and The Humber	16	10,538	0.15% (0.09% , 0.25%)	0.17% (0.10% , 0.29%)	70	9,550	0.73% (0.57% , 0.93%)	0.88% (0.67% , 1.15%)
	East Midlands	12	9,145	0.13% (0.07% , 0.23%)	0.19% (0.10% , 0.36%)	47	8,425	0.56% (0.41% , 0.74%)	0.65% (0.47% , 0.89%)
	West Midlands	12	10,993	0.11% (0.06% , 0.19%)	0.11% (0.06% , 0.20%)	49	9,842	0.50% (0.37% , 0.66%)	0.56% (0.40% , 0.77%)
	East of England	17	12,388	0.14% (0.08% , 0.22%)	0.14% (0.08% , 0.24%)	42	11,210	0.37% (0.27% , 0.51%)	0.41% (0.30% , 0.58%)
	London	22	16,966	0.13% (0.08% , 0.20%)	0.13% (0.08% , 0.20%)	111	14,598	0.76% (0.63% , 0.92%)	0.94% (0.76% , 1.16%)
	South West	7	11,779	0.06% (0.02% , 0.12%)	0.05% (0.02% , 0.12%)	39	10,867	0.36% (0.26% , 0.49%)	0.43% (0.30% , 0.61%)

* For categories other than age and region, we present weighted prevalence if the number of positives in a category is 10 or more.

Table S3b. Unweighted (95% confidence interval) and weighted (95% credible interval) prevalence of swab-positivity for employment type, ethnic group, and household size for round 12 and round 13.

Variable	Category	Round 12 Positive	Round 12 Total	Round 12 Unweighted Prevalence	Round 12 Weighted Prevalence*	Round 13 Positive	Round 13 Total	Round 13 Unweighted Prevalence	Round 13 Weighted Prevalence*
Employment type	Health care or care home worker	6	7,955	0.08% (0.03% , 0.16%)	NA (NA , NA)	42	7,415	0.57% (0.41% , 0.76%)	0.64% (0.46% , 0.89%)
	Other essential/key worker	17	16,663	0.10% (0.06% , 0.16%)	0.12% (0.07% , 0.22%)	89	14,327	0.62% (0.50% , 0.76%)	0.67% (0.53% , 0.85%)
	Other worker	73	41,503	0.18% (0.14% , 0.22%)	0.20% (0.16% , 0.26%)	209	37,818	0.55% (0.48% , 0.63%)	0.65% (0.55% , 0.75%)
	Not full-time, part-time, or self-employed	36	40,639	0.09% (0.06% , 0.12%)	0.10% (0.07% , 0.15%)	157	36,633	0.43% (0.36% , 0.50%)	0.54% (0.45% , 0.65%)
	Unknown	3	2,151	0.14% (0.03% , 0.41%)	NA (NA , NA)	30	2,040	1.47% (0.99% , 2.09%)	1.27% (0.86% , 1.86%)
Ethnic group	White	104	95,019	0.11% (0.09% , 0.13%)	0.13% (0.11% , 0.16%)	427	85,518	0.50% (0.45% , 0.55%)	0.59% (0.53% , 0.65%)
	Asian	15	6,386	0.23% (0.13% , 0.39%)	0.28% (0.16% , 0.51%)	43	5,772	0.75% (0.54% , 1.00%)	0.83% (0.59% , 1.16%)
	Black	4	2,289	0.17% (0.05% , 0.45%)	NA (NA , NA)	21	2,036	1.03% (0.64% , 1.57%)	1.21% (0.75% , 1.93%)
	Mixed	3	1,998	0.15% (0.03% , 0.44%)	NA (NA , NA)	19	1,823	1.04% (0.63% , 1.62%)	0.98% (0.62% , 1.57%)
	Other	3	1,257	0.24% (0.05% , 0.70%)	NA (NA , NA)	7	1,063	0.66% (0.27% , 1.35%)	0.86% (0.40% , 1.87%)
	Unknown	6	1,962	0.31% (0.11% , 0.66%)	NA (NA , NA)	10	2,021	0.49% (0.24% , 0.91%)	0.40% (0.21% , 0.77%)
Household size	1	10	16,885	0.06% (0.03% , 0.11%)	0.07% (0.03% , 0.14%)	58	15,556	0.37% (0.28% , 0.48%)	0.44% (0.32% , 0.61%)
	2	37	41,682	0.09% (0.06% , 0.12%)	0.09% (0.07% , 0.13%)	136	37,287	0.36% (0.31% , 0.43%)	0.44% (0.36% , 0.53%)
	3	29	19,196	0.15% (0.10% , 0.22%)	0.20% (0.14% , 0.31%)	98	17,202	0.57% (0.46% , 0.69%)	0.61% (0.49% , 0.76%)
	4	34	20,955	0.16% (0.11% , 0.23%)	0.17% (0.12% , 0.26%)	148	19,328	0.77% (0.65% , 0.90%)	0.81% (0.67% , 0.97%)
	5	15	7,050	0.21% (0.12% , 0.35%)	0.24% (0.14% , 0.41%)	59	6,287	0.94% (0.72% , 1.21%)	1.01% (0.76% , 1.34%)
	6+	10	3,143	0.32% (0.15% , 0.58%)	0.34% (0.17% , 0.68%)	28	2,573	1.09% (0.72% , 1.57%)	1.35% (0.90% , 2.01%)

* For categories other than age and region, we present weighted prevalence if the number of positives in a category is 10 or more.

Table S3c. Unweighted (95% confidence interval) and weighted (95% credible interval) prevalence of swab-positivity for COVID case contact status, symptom status, neighbourhood deprivation and vaccination status for round 12 and round 13.

Variable	Category	Round 12 Positive	Round 12 Total	Round 12 Unweighted Prevalence	Round 12 Weighted Prevalence*	Round 13 Positive	Round 13 Total	Round 13 Unweighted Prevalence	Round 13 Weighted Prevalence*
COVID case contact	No	70	90,682	0.08% (0.06% , 0.10%)	0.09% (0.07% , 0.12%)	248	78,814	0.31% (0.28% , 0.36%)	0.39% (0.34% , 0.45%)
	Yes, contact with a confirmed/tested COVID-19 case	21	438	4.79% (2.99% , 7.24%)	4.46% (2.77% , 7.10%)	165	2,843	5.80% (4.97% , 6.73%)	5.59% (4.75% , 6.57%)
	Yes, contact with a suspected COVID-19 case	3	229	1.31% (0.27% , 3.78%)	NA (NA , NA)	24	1,075	2.23% (1.44% , 3.30%)	2.51% (1.60% , 3.91%)
	Unknown	41	17,562	0.23% (0.17% , 0.32%)	0.27% (0.19% , 0.39%)	90	15,501	0.58% (0.47% , 0.71%)	0.66% (0.52% , 0.84%)
Symptom status	Classic COVID symptoms	32	2,640	1.21% (0.83% , 1.71%)	1.52% (1.04% , 2.22%)	174	2,936	5.93% (5.10% , 6.84%)	6.46% (5.48% , 7.61%)
	Other symptoms	9	10,841	0.08% (0.04% , 0.16%)	NA (NA , NA)	62	9,859	0.63% (0.48% , 0.81%)	0.73% (0.55% , 0.95%)
	No symptoms	53	77,932	0.07% (0.05% , 0.09%)	0.08% (0.06% , 0.10%)	202	70,011	0.29% (0.25% , 0.33%)	0.34% (0.29% , 0.39%)
	Unknown	41	17,498	0.23% (0.17% , 0.32%)	0.28% (0.19% , 0.39%)	89	15,427	0.58% (0.46% , 0.71%)	0.66% (0.52% , 0.84%)
Deprivation	1 Most deprived	27	12,916	0.21% (0.14% , 0.30%)	0.25% (0.17% , 0.37%)	84	11,541	0.73% (0.58% , 0.90%)	0.82% (0.65% , 1.04%)
	2	24	18,796	0.13% (0.08% , 0.19%)	0.15% (0.10% , 0.24%)	102	16,794	0.61% (0.50% , 0.74%)	0.68% (0.55% , 0.84%)
	3	24	22,762	0.11% (0.07% , 0.16%)	0.10% (0.07% , 0.16%)	110	20,556	0.54% (0.44% , 0.64%)	0.60% (0.48% , 0.73%)
	4	34	25,891	0.13% (0.09% , 0.18%)	0.14% (0.10% , 0.21%)	118	23,284	0.51% (0.42% , 0.61%)	0.59% (0.48% , 0.72%)
	5 Least deprived	26	28,546	0.09% (0.06% , 0.13%)	0.10% (0.07% , 0.16%)	113	26,058	0.43% (0.36% , 0.52%)	0.48% (0.39% , 0.59%)
Vaccination status	Unkown	33	16,174	0.20% (0.14% , 0.29%)	0.23% (0.15% , 0.35%)	64	14,136	0.45% (0.35% , 0.58%)	0.52% (0.39% , 0.70%)
	Unvaccinated	51	22,760	0.22% (0.17% , 0.29%)	0.24% (0.18% , 0.33%)	178	15,135	1.18% (1.01% , 1.36%)	1.21% (1.03% , 1.41%)
	Vaccinated - 1 dose	20	18,674	0.11% (0.07% , 0.17%)	0.11% (0.07% , 0.18%)	77	9,675	0.80% (0.63% , 0.99%)	0.80% (0.63% , 1.03%)
	Vaccinated - 2 doses**	30	48,413	0.06% (0.04% , 0.09%)	0.07% (0.05% , 0.10%)	197	55,962	0.35% (0.30% , 0.40%)	0.40% (0.34% , 0.48%)
	Dose number not reported	1	2,890	0.03% (0.00% , 0.19%)	0.05% (0.01% , 0.33%)	11	3,325	0.33% (0.17% , 0.59%)	0.44% (0.22% , 0.86%)

* For categories other than age and region, we present weighted prevalence if the number of positives in a category is 10 or more.
** Small number reporting 3 doses have been included in this group (<30 participants)

Table S4. Comparison of self-reported vaccination status and swab-positivity for participants who did and did not consent to data linkage (odds ratios and 95% confidence intervals).

	Linked			Unlinked		
Vaccination status	Negative	Positive	Odds ratio	Negative	Positive	Odds ratio
Unvaccinated	2174	20	Reference	400	8	Reference
Vaccinated (1 dose)	8048	67	0.90 (0.55 , 1.49)	1419	9	0.32 (0.12 , 0.83)
Vaccinated (2 or more doses)	30371	133	0.48 (0.30 , 0.76)	4132	12	0.15 (0.06 , 0.36)
Vaccinated (unknown doses)	1330	7	0.57 (0.24 , 1.36)	187	2	0.53 (0.11 , 2.54)
Vaccine status not known	7730	43	0.60 (0.35 , 1.03)	1359	6	0.22 (0.08 , 0.64)
Total	49653	270		7497	37	

Table S5. Unweighted prevalence (95% confidence interval) of infection by self-reported vaccine status and self-reported prior infection status.

		Round 12			Round 13		
Vaccination Status	Previous infection status	Positive	Total	Unweighted Prevalence	Positive	Total	Unweighted Prevalence
Status not known	No previous infection	0	980	0.00% (0.00% , 0.38%)	1	765	0.13% (0.00% , 0.73%)
	Previous infection within 28 days	0	1	0.00% (0.00% , 97.50%)	2	4	50.00% (6.76% , 93.24%)
	Previous infection greater than 28 days	0	77	0.00% (0.00% , 4.68%)	0	81	0.00% (0.00% , 4.45%)
	Previous infection with unknown time	0	7	0.00% (0.00% , 40.96%)	0	7	0.00% (0.00% , 40.96%)
	Suspected previous infection	0	141	0.00% (0.00% , 2.58%)	1	172	0.58% (0.01% , 3.20%)
	Status not known	33	14968	0.22% (0.15% , 0.31%)	60	13107	0.46% (0.35% , 0.59%)
Unvaccinated	No previous infection	26	17266	0.15% (0.10% , 0.22%)	72	11168	0.64% (0.50% , 0.81%)
	Previous infection within 28 days	11	20	55.00% (31.53% , 76.94%)	59	80	73.75% (62.71% , 82.96%)
	Previous infection greater than 28 days	1	912	0.11% (0.00% , 0.61%)	4	453	0.88% (0.24% , 2.25%)
	Previous infection with unknown time	0	1	0.00% (0.00% , 97.50%)	1	3	33.33% (0.84% , 90.57%)
	Suspected previous infection	5	1958	0.26% (0.08% , 0.59%)	12	1028	1.17% (0.60% , 2.03%)
	Status not known	8	2603	0.31% (0.13% , 0.60%)	30	2403	1.25% (0.84% , 1.78%)
Vaccinated - 1 dose	No previous infection	8	15012	0.05% (0.02% , 0.11%)	27	7701	0.35% (0.23% , 0.51%)
	Previous infection within 28 days	6	14	42.86% (17.66% , 71.14%)	35	56	62.50% (48.55% , 75.08%)
	Previous infection greater than 28 days	2	1180	0.17% (0.02% , 0.61%)	2	568	0.35% (0.04% , 1.27%)
	Previous infection with unknown time	0	0	NA (0.00% , 100.00%)	0	1	0.00% (0.00% , 97.50%)
	Suspected previous infection	4	2468	0.16% (0.04% , 0.41%)	13	1349	0.96% (0.51% , 1.64%)
	Status not known	0	0	NA (0.00% , 100.00%)	0	0	NA (0.00% , 100.00%)
Vaccinated - 2 doses *	No previous infection	20	41755	0.05% (0.03% , 0.07%)	111	47483	0.23% (0.19% , 0.28%)
	Previous infection within 28 days	4	9	44.44% (13.70% , 78.80%)	51	99	51.52% (41.25% , 61.68%)
	Previous infection greater than 28 days	5	2299	0.22% (0.07% , 0.51%)	7	2813	0.25% (0.10% , 0.51%)
	Previous infection with unknown time	0	7	0.00% (0.00% , 40.96%)	0	3	0.00% (0.00% , 70.76%)
	Suspected previous infection	1	4342	0.02% (0.00% , 0.13%)	28	5563	0.50% (0.33% , 0.73%)
	Status not known	0	1	0.00% (0.00% , 97.50%)	0	1	0.00% (0.00% , 97.50%)
Vaccinated - dose not known	No previous infection	1	2680	0.04% (0.00% , 0.21%)	8	3041	0.26% (0.11% , 0.52%)
	Previous infection within 28 days	0	0	NA (0.00% , 100.00%)	3	3	100.00% (29.24% , 100.00%)
	Previous infection greater than 28 days	0	15	0.00% (0.00% , 21.80%)	0	12	0.00% (0.00% , 26.46%)
	Previous infection with unknown time	0	0	NA (0.00% , 100.00%)	0	0	NA (0.00% , 100.00%)
	Suspected previous infection	0	195	0 (0.00% , 1.87%)	0	269	0.00% (0.00% , 1.36%)
	Status not known	0	0	NA (0.00% , 100.00%)	0	0	NA (0.00% , 100.00%)

* Small number reporting 3 doses have been included in this group (<30 participants).

Table S6. Unweighted (95% confidence interval) and weighted (95% credible interval) prevalence of infection by vaccine and contact status for rounds 12 and 13 of REACT-1.

Vaccination Status	Contact	Round 12				Round 13			
		Positive	Total	Unweighted Prevalence	Weighted prevalence**	Positive	Total	Unweighted Prevalence	Weighted prevalence**
Not vaccinated	Yes, confirmed/tested COVID-19 case	13	194	6.70% (3.62% , 11.19%)	6.12% (3.33% , 10.97%)	74	992	7.46% (5.90% , 9.27%)	7.23% (5.71% , 9.12%)
	Yes, suspected COVID-19 case	2	98	2.04% (0.25% , 7.18%)	NA (NA , NA)	8	315	2.54% (1.10% , 4.94%)	NA (NA , NA)
	No	28	19865	0.14% (0.09% , 0.20%)	0.15% (0.10% , 0.22%)	66	11429	0.58% (0.45% , 0.73%)	0.62% (0.48% , 0.81%)
	Not known	8	2603	0.31% (0.13% , 0.60%)	NA (NA , NA)	30	2399	1.25% (0.85% , 1.78%)	1.28% (0.86% , 1.88%)
Vaccinated - 1 dose	Yes, confirmed/tested COVID-19 case	4	78	5.13% (1.41% , 12.61%)	3.83% (1.22% , 11.36%)	36	446	8.07% (5.72% , 11.00%)	5.86% (4.14% , 8.23%)
	Yes, suspected COVID-19 case	1	32	3.13% (0.08% , 16.22%)	NA (NA , NA)	4	152	2.63% (0.72% , 6.60%)	NA (NA , NA)
	No	15	18564	0.08% (0.05% , 0.13%)	0.09% (0.05% , 0.15%)	37	9077	0.41% (0.29% , 0.56%)	0.49% (0.35% , 0.70%)
	Not known	0	0	NA (0.00% , 100.00%)	NA (NA , NA)	NA	NA	NA (NA , NA)	NA (NA , NA)
Vaccinated - 2 doses *	Yes, confirmed/tested COVID-19 case	4	144	2.78% (0.76% , 6.96%)	NA (NA , NA)	51	1312	3.89% (2.91% , 5.08%)	3.84% (2.81% , 5.21%)
	Yes, suspected COVID-19 case	0	94	0.00% (0.00% , 3.85%)	NA (NA , NA)	12	559	2.15% (1.11% , 3.72%)	2.21% (1.22% , 3.99%)
	No	26	48174	0.05% (0.04% , 0.08%)	0.06% (0.04% , 0.09%)	134	54091	0.25% (0.21% , 0.29%)	0.30% (0.24% , 0.36%)
	Not known	0	1	0.00% (0.00% , 97.50%)	NA (NA , NA)	NA	NA	NA (NA , NA)	NA (NA , NA)
Vaccinated - dose not known	Yes, confirmed/tested COVID-19 case	0	11	0.00% (0.00% , 28.49%)	NA (NA , NA)	2	50	4.00% (0.49% , 13.71%)	NA (NA , NA)
	Yes, suspected COVID-19 case	0	3	0.00% (0.00% , 70.76%)	NA (NA , NA)	0	27	0.00% (0.00% , 12.77%)	NA (NA , NA)
	No	1	2876	0.03% (0.00% , 0.19%)	NA (NA , NA)	9	3248	0.28% (0.13% , 0.53%)	NA (NA , NA)
	Not known	NA	NA	NA (NA , NA)	NA (NA , NA)	NA	NA	NA (NA , NA)	NA (NA , NA)
Vaccine status not known	Yes, confirmed/tested COVID-19 case	0	11	0.00% (0.00% , 28.49%)	NA (NA , NA)	2	43	4.65% (0.57% , 15.81%)	NA (NA , NA)
	Yes, suspected COVID-19 case	0	2	0.00% (0.00% , 84.19%)	NA (NA , NA)	0	22	0.00% (0.00% , 15.44%)	NA (NA , NA)
	No	0	1203	0.00% (0.00% , 0.31%)	NA (NA , NA)	2	969	0.21% (0.03% , 0.74%)	NA (NA , NA)
	Not known	33	14958	0.22% (0.15% , 0.31%)	0.25% (0.17% , 0.37%)	60	13102	0.46% (0.35% , 0.59%)	0.52% (0.38% , 0.70%)

*Small number reporting 3 doses have been included in this group (<30 participants).

** We present weighted prevalence if the number of positives in a category is 10 or more.

Table S7. Median N-gene Ct values between individuals with different vaccine status, ages 18-64 years, round 13. Estimates have been calculated for all data available and subsets of data with lower N-gene Ct values.

Data	Vaccine status	Negatives	Positives	Median N-gene Ct value*	P-value**
All data	Not vaccinated	2574	28	23.1 (20.3 , 25.8)	ref
	1 vaccine doses	9467	76	27.4 (24.8 , 30.0)	0.04
	2 vaccine doses	34484	145	27.6 (25.5 , 29.7)	0.01
	Vaccine doses not known	1517	9	29.7 (25.6 , 33.7)	0.01
N-gene Ct <35	Not vaccinated	2574	27	22.9 (20.8 , 25.0)	ref
	1 vaccine doses	9467	68	25.9 (23.6 , 28.2)	0.09
	2 vaccine doses	34484	118	25.5 (23.9 , 27.1)	0.09
	Vaccine doses not known	1517	7	28.7 (26.5 , 31.0)	0.05
N-gene Ct <33	Not vaccinated	2574	26	22.9 (20.4 , 25.5)	ref
	1 vaccine doses	9467	62	25.2 (22.6 , 27.8)	0.15
	2 vaccine doses	34484	99	24.3 (22.5 , 26.1)	0.41
	Vaccine doses not known	1517	6	27.7 (24.3 , 31.2)	0.10

* 95% Confidence intervals in the median were estimated using quantile regression

**P-values were calculated using the two-sided Wilcoxon two-sample test (also known as Mann Whitney-U) and are relative to the distribution of Ct values in individuals who are not vaccinated

Table S8. Estimates of regional growth rates, doubling times and reproduction numbers (95% credible intervals) for round 13, and round 12 to round 13.

Round	Region	Growth rate	R	Probability R>1	Halving (-) / Doubling (+) time
12 and 13	East Midlands	0.038 (0.024 , 0.055)	1.26 (1.16 , 1.38)	>0.99	18.0 (29.5 , 12.6)
	West Midlands	0.048 (0.031 , 0.066)	1.33 (1.21 , 1.47)	>0.99	14.5 (22.3 , 10.5)
	East of England	0.032 (0.017 , 0.048)	1.21 (1.11 , 1.33)	>0.99	21.9 (41.9 , 14.4)
	London	0.052 (0.040 , 0.064)	1.36 (1.28 , 1.46)	>0.99	13.4 (17.2 , 10.8)
	North West	0.031 (0.021 , 0.043)	1.21 (1.14 , 1.29)	>0.99	22.1 (33.3 , 16.2)
	North East	0.050 (0.029 , 0.076)	1.35 (1.19 , 1.55)	>0.99	13.8 (24.0 , 9.1)
	South East	0.029 (0.017 , 0.042)	1.19 (1.11 , 1.29)	>0.99	24.3 (42.0 , 16.6)
	South West	0.058 (0.037 , 0.083)	1.41 (1.25 , 1.61)	>0.99	11.9 (18.8 , 8.3)
	Yorkshire **	0.046 (0.033 , 0.061)	1.32 (1.22 , 1.43)	>0.99	14.9 (21.1 , 11.3)
13	East Midlands	0.054 (-0.009 , 0.117)	1.38 (0.94 , 1.91)	0.95	12.8 (* , 5.9)
	West Midlands	0.063 (0.001 , 0.127)	1.45 (1.01 , 2.00)	0.98	10.9 (* , 5.5)
	East of England	0.014 (-0.055 , 0.081)	1.09 (0.69 , 1.59)	0.66	* (-12.7 , 8.6)
	London	-0.017 (-0.056 , 0.022)	0.90 (0.68 , 1.15)	0.20	-41.5 (-12.3 , 31.6)
	North West	0.045 (-0.003 , 0.092)	1.31 (0.98 , 1.69)	0.97	15.4 (* , 7.5)
	North East	0.031 (-0.051 , 0.112)	1.21 (0.71 , 1.86)	0.77	22.1 (-13.6 , 6.2)
	South East	0.017 (-0.042 , 0.074)	1.11 (0.75 , 1.54)	0.71	41.8 (-16.5 , 9.3)
	South West	0.049 (-0.020 , 0.120)	1.34 (0.88 , 1.93)	0.92	14.3 (-34.3 , 5.8)
	Yorkshire **	0.042 (-0.009 , 0.093)	1.29 (0.95 , 1.69)	0.95	16.4 (-79.4 , 7.5)

* Doubling/Halving time had an estimated magnitude greater than 50 days and so represented approximately constant prevalence

** Yorkshire and The Humber

Table S9. Odds ratios (95% confidence intervals) from multivariable logistic regression models of positivity for rounds 12 and round 13 of REACT-1.

Variable	Category	Round 12**		Round 13 **	
		Adjusted for age and sex	Mutually adjusted*	Adjusted for age and sex	Mutually adjusted*
Sex	Male	Ref	Ref	Ref	Ref
	Female	1.13 (0.80,1.59)	1.34 (0.93,1.92)	0.82 (0.69,0.97)	0.80 (0.67,0.96)
Age Group	5-12	2.73 (1.46,5.10)	2.61 (1.32,5.17)	1.60 (1.15,2.23)	1.71 (1.22,2.41)
	13-17	1.34 (0.61,2.96)	1.87 (0.76,4.63)	2.58 (1.89,3.53)	2.82 (1.90,4.18)
	18-24	2.33 (1.10,4.92)	2.57 (1.18,5.64)	2.50 (1.73,3.62)	2.61 (1.78,3.83)
	25-34	0.95 (0.44,2.06)	1.12 (0.50,2.47)	1.12 (0.79,1.60)	1.14 (0.79,1.63)
	35-44	Ref	Ref	Ref	Ref
	45-54	1.03 (0.55,1.96)	1.22 (0.62,2.39)	0.76 (0.55,1.05)	0.76 (0.54,1.06)
	55-64	0.60 (0.30,1.20)	0.82 (0.39,1.72)	0.50 (0.36,0.71)	0.54 (0.38,0.78)
	65+	0.54 (0.28,1.04)	0.80 (0.36,1.75)	0.36 (0.26,0.50)	0.40 (0.27,0.59)
Region	North East	1.12 (0.45,2.78)	1.14 (0.45,2.86)	1.80 (1.14,2.84)	1.81 (1.14,2.88)
	North West	1.54 (0.85,2.81)	1.40 (0.75,2.63)	2.00 (1.43,2.79)	1.73 (1.21,2.47)
	Yorkshire and The Humber	1.37 (0.72,2.63)	1.39 (0.71,2.70)	2.12 (1.50,2.99)	2.05 (1.43,2.92)
	East Midlands	1.18 (0.58,2.39)	1.20 (0.58,2.47)	1.63 (1.11,2.39)	1.62 (1.10,2.39)
	West Midlands	0.99 (0.48,2.01)	0.77 (0.36,1.66)	1.42 (0.97,2.07)	1.36 (0.92,2.00)
	East of England	1.24 (0.65,2.34)	1.06 (0.54,2.11)	1.05 (0.71,1.56)	1.03 (0.69,1.55)
	London	1.07 (0.59,1.95)	0.84 (0.44,1.60)	1.92 (1.41,2.64)	1.69 (1.20,2.36)
	South East	Ref	Ref	Ref	Ref
	South West	0.55 (0.23,1.30)	0.58 (0.25,1.38)	1.05 (0.70,1.57)	0.97 (0.64,1.49)
Key Worker Status	HCW/CHW	0.37 (0.16,0.85)	0.37 (0.16,0.87)	1.00 (0.71,1.40)	0.99 (0.70,1.39)
	Key worker (other)	0.52 (0.30,0.88)	0.55 (0.32,0.94)	1.07 (0.83,1.38)	1.08 (0.83,1.39)
	Other worker	Ref	Ref	Ref	Ref
	Not FT, PT, SE	0.58 (0.36,0.92)	0.60 (0.38,0.95)	0.88 (0.69,1.12)	0.87 (0.68,1.11)
Ethnicity	White	Ref	Ref	Ref	Ref
	Asian	1.69 (0.97,2.93)	1.52 (0.84,2.76)	1.08 (0.78,1.48)	1.01 (0.72,1.41)
	Black	1.27 (0.47,3.47)	1.26 (0.45,3.55)	1.57 (1.01,2.45)	1.48 (0.93,2.35)
	Mixed	0.91 (0.29,2.91)	0.95 (0.30,3.05)	1.27 (0.80,2.03)	1.00 (0.58,1.73)
	Other	1.85 (0.59,5.87)	1.82 (0.57,5.87)	1.09 (0.51,2.30)	0.87 (0.39,1.98)
Household Size	1-2 People	Ref	Ref	Ref	Ref
	3-5 People	1.28 (0.81,2.01)	1.19 (0.75,1.89)	0.94 (0.75,1.17)	0.95 (0.76,1.20)
	6+ People	2.07 (0.97,4.41)	1.74 (0.78,3.88)	1.19 (0.78,1.83)	1.14 (0.72,1.80)
Deprivation Index Quintile	1 - Most Deprived	2.06 (1.20,3.54)	1.94 (1.08,3.51)	1.48 (1.12,1.97)	1.29 (0.95,1.75)
	2	1.32 (0.76,2.31)	1.39 (0.78,2.51)	1.31 (1.00,1.71)	1.15 (0.86,1.53)
	3	1.15 (0.66,2.00)	1.11 (0.62,2.01)	1.20 (0.92,1.56)	1.12 (0.85,1.48)
	4	1.45 (0.87,2.42)	1.55 (0.91,2.63)	1.17 (0.90,1.52)	1.13 (0.86,1.47)
	5 - Least Deprived	Ref	Ref	Ref	Ref

* Odds ratios mutually adjusted for all variables shown.

**The sample size for variables in round 12 is 104,844 and in round 13 is 94,223

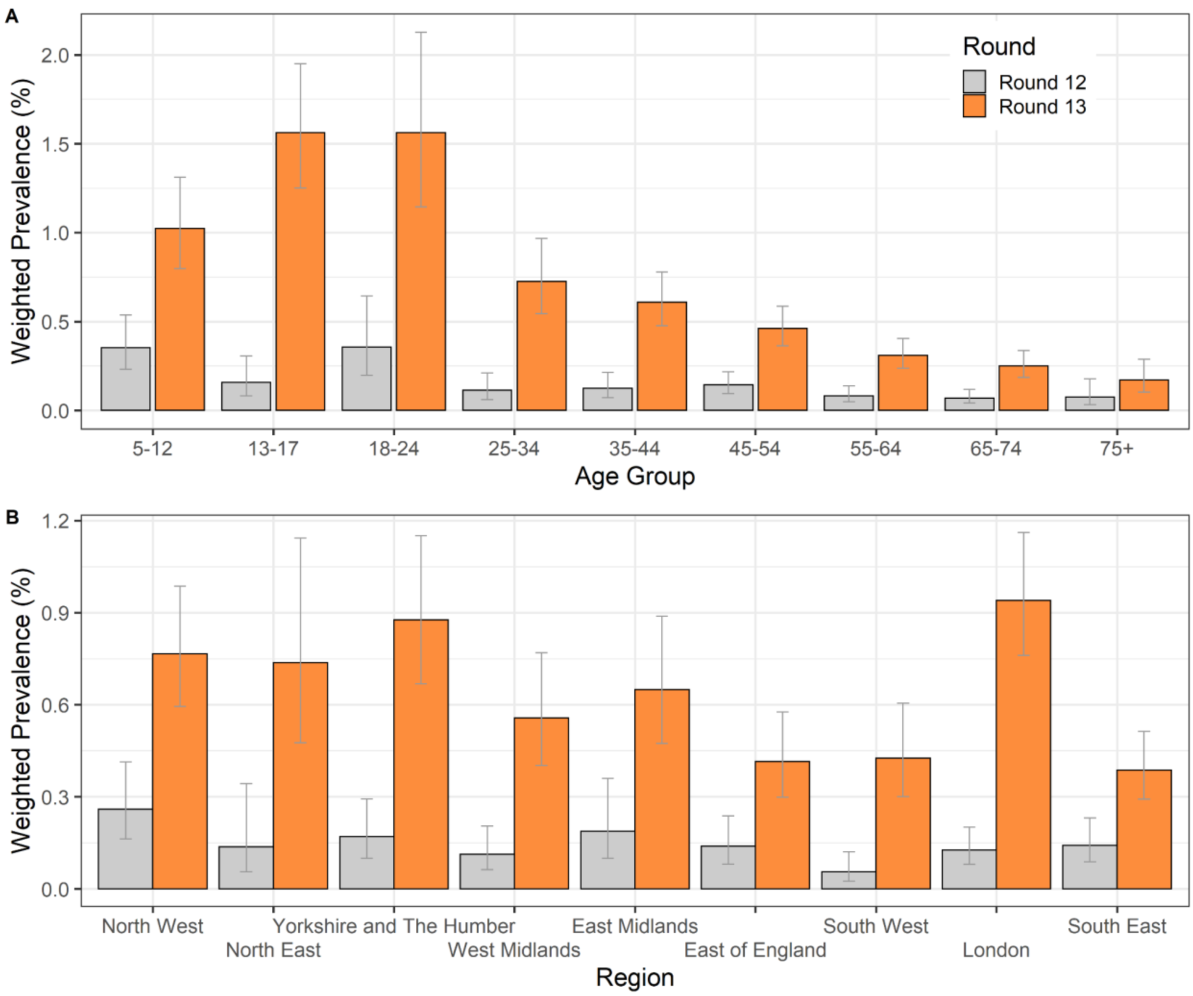

Figure S1. Weighted prevalence of swab-positivity by age group and by region for round 12 and round 13 complete. Bars show 95% credible intervals. (A) Weighted prevalence of swab-positivity by age group. (B) Weighted prevalence of swab-positivity by region.

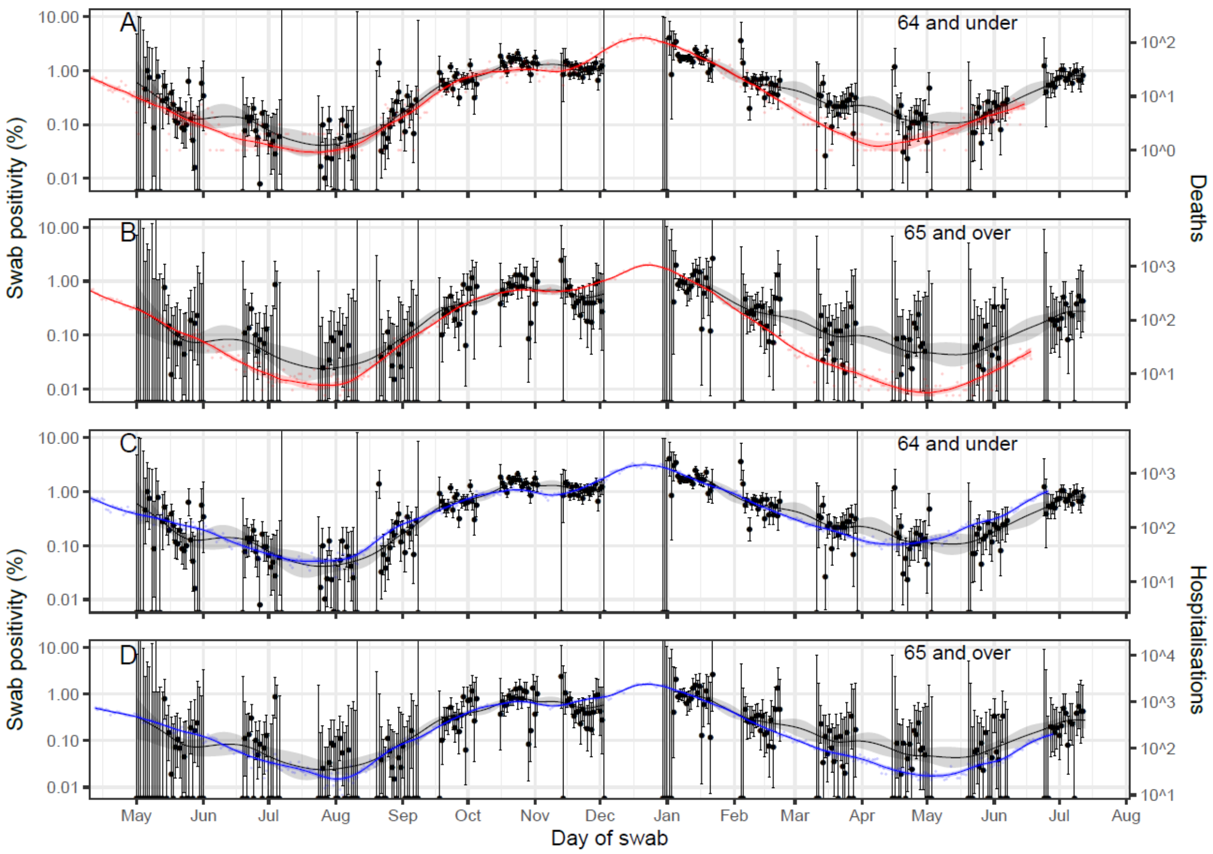

Figure S2. A comparison of daily deaths and hospitalisations to swab positivity as measured by REACT-1, by age group. Daily swab positivity for all 12 rounds of the REACT-1 study (black points with 95% confidence intervals, left hand y-axis) with P-spline estimates for swab positivity (solid black line, shaded area is 95% confidence interval) for (A, C) those aged under 64 years and (B,D) those aged 65 years and over. **(A)** Daily deaths for those aged 64 and under in England (red points, right hand y-axis) and corresponding P-spline model estimates for the expected number of deaths (solid red line, shaded area is 95% confidence interval, right hand y-axis). Daily deaths have been shifted by 29 (29, 29) days backwards in time along the x-axis. The two y-axes have been scaled using the best-fit population adjusted scaling parameter 0.0065 (0.0064, 0.0067). **(B)** Daily deaths for those aged 65 and over in England (red points, right hand y-axis) and corresponding P-spline model estimates for the expected number of deaths (solid red line, shaded area is 95% confidence interval, right hand y-axis). Daily deaths have been shifted by 26 (25, 28) days backwards in time along the x-axis. The two y-axes have been scaled using the best-fit scaling parameter 0.51 (0.48, 0.54). **(C)** Daily hospitalisations for those aged 64 and under in England (blue points, right hand y-axis) and corresponding P-spline model estimates for the expected number of hospitalisations (solid blue line, shaded area is 95% confidence interval, right hand y-axis). Daily hospitalisations have been shifted by 21 (20, 21) days backwards in time along the x-axis. The two y-axes have been scaled using the best-fit scaling parameter 0.101 (0.098, 0.103). **(D)** Daily hospitalisations for those aged 65 and over in England (blue points, right hand y-axis) and corresponding P-spline model estimates for the expected number of hospitalisations (solid blue line, shaded area is 95% confidence interval, right hand y-axis). Daily hospitalisations have been shifted by 19 (17, 20) days backwards in time along the x-axis. The two y-axes have been scaled using the best-fit scaling parameter 1.41 (1.33, 1.50).

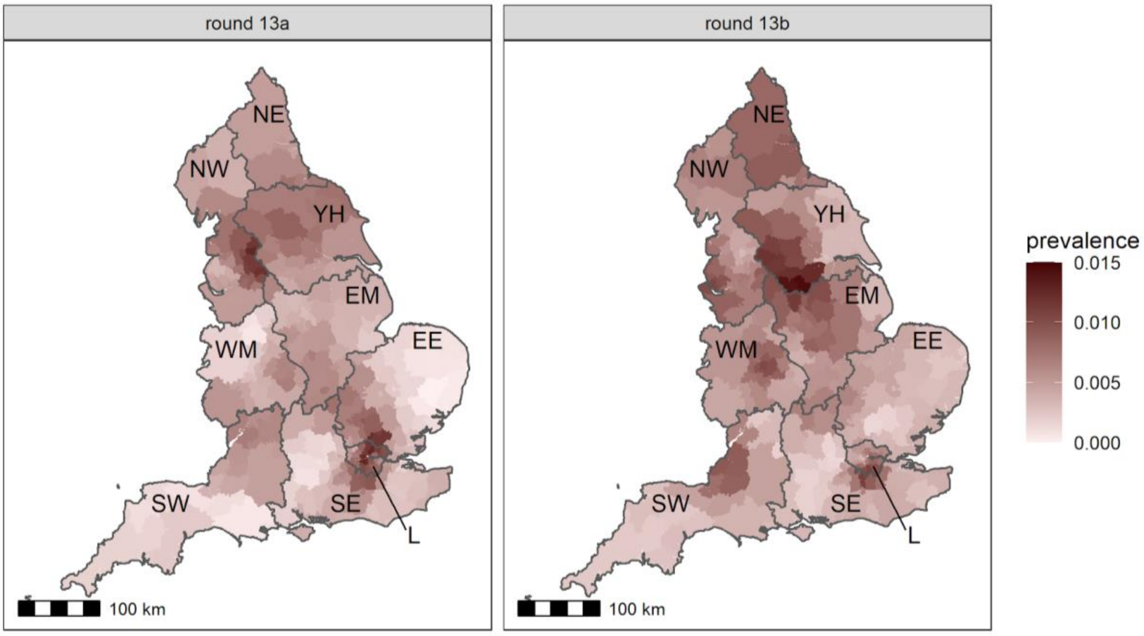

Figure S3. Neighbourhood smoothed average prevalence by lower tier local area for (A) round 13a and (B) round 13b. Neighbourhood prevalence calculated from nearest neighbours (the median number of neighbours within 30 km in the study). Average neighbourhood prevalence displayed for individual lower-tier local authorities. Regions: NE = North East, NW = North West, YH = Yorkshire and The Humber, EM = East Midlands, WM = West Midlands, EE = East of England, L = London, SE = South East, SW = South West.
